# A nostoxanthin-producing bacterium, *Sphingomonas nostoxanthinifaciens* sp. nov., alleviates the salt stress of *Arabidopsis* seedlings by scavenging of reactive oxygen species

**DOI:** 10.3389/fmicb.2023.1101150

**Published:** 2023-02-10

**Authors:** Lingmin Jiang, Jiyoon Seo, Yuxin Peng, Doeun Jeon, Ju Huck Lee, Cha Young Kim, Jiyoung Lee

**Affiliations:** Biological Resource Center, Korean Collection for Type Cultures (KCTC), Korea Research Institute of Bioscience and Biotechnology (KRIBB), Jeongeup, Republic of Korea

**Keywords:** polyphasic taxonomy, *Abies koreana*, phylogeny, orthologous average nucleotide identity, whole-genome annotation, antiSMASH

## Abstract

A novel, nostoxanthin-producing, endophytic bacterium, designated as AK-PDB1-5^T^, was isolated from the needle-like leaves of the Korean fir (*Abies koreana* Wilson) collected from Mt. Halla in Jeju, South Korea. A 16S rRNA sequence comparison indicated that the closest phylogenetic neighbors were *Sphingomonas crusticola* MIMD3^T^ (95.6%) and *Sphingomonas jatrophae* S5-249^T^ (95.3%) of the family *Sphingomonadaceae*. Strain AK-PDB1-5^T^ had a genome size of 4,298,284 bp with a 67.8% G + C content, and digital DNA–DNA hybridization and OrthoANI values with the most closely related species of only 19.5–21% and 75.1–76.8%, respectively. Cells of the strain AK-PDB1-5^T^ were Gram-negative, short rods, oxidase- and catalase-positive. Growth occurred at pH 5.0–9.0 (optimum pH 8.0) in the absence of NaCl at 4–37°C (optimum 25–30°C). Strain AK-PDB1-5^T^ contained C_14:0_ 2OH_,_ C_16:0_ and summed feature 8 as the major cellular fatty acids (> 10%), while sphingoglycolipid, phosphatidylethanolamine, phosphatidylglycerol, phospholipids and lipids were found to be the major polar lipids. The strain produces a yellow carotenoid pigment; natural products prediction *via* AntiSMASH tool found zeaxanthin biosynthesis clusters in the entire genome. Biophysical characterization by ultraviolet–visible absorption spectroscopy and ESI-MS studies confirmed the yellow pigment was nostoxanthin. In addition, strain AK-PDB1-5^T^ was found significantly promote Arabidopsis seedling growth under salt conditions by reducing reactive oxygen species (ROS). Based on the polyphasic taxonomic analysis results, strain AK-PDB1-5^T^ was determined to be a novel species in the genus *Sphingomonas* with the proposed name *Sphingomonas nostoxanthinifaciens* sp. nov. The type strain is AK-PDB1-5^T^ (= KCTC 82822^T^ = CCTCC AB 2021150^T^).

## 1. Introduction

The genus *Sphingomonas* belonging to the family *Sphingomonadaceae* of the class *Alphaproteobacteria* was first proposed by Yabuuchi et al. (1990). The type strain is *Sphingomonadaceae paucimobilis* VKM B-2241^T^, and four other members were proposed at the same time (Holmes et al., 1977). The genus *Sphingomonas* has been divided into four clusters based on phylogenetic and chemotaxonomic analysis in 2001, and three new genera *Sphingobium*, *Novosphingobium*, and *Sphingopyxis* were proposed in addition to the genus *Sphingomonas sensu stricto* (Takeuchi et al., 2001). As of August 2022, this genus contains 146 published species.^1^ The common cellular characteristics of the genus *Sphingomonas* are Gram-negative, a yellow or white-color, rod shape, strictly aerobic, motile or non-motile, and non-fermentative. Q-10 is the predominant isoprenoid quinone in this genus, while the major fatty acids are C_16:0_, C_14:0_ 2OH, as well as summed feature 8 (comprising C_18:1_ω7c and/or C_18:1_ω6c). Species in this genus have been isolated from various sources, including the soil (Kim et al., 2014), water, air (Kim et al., 2014; Xue et al., 2018), plant endophytes, rice paddy and noni, and a medical clinic (Busse et al., 2005). Some *Sphingomonas* species such as *Sphingomonas laterariae* and *Sphingomonas wittichii* have been found in contaminated environments, probably due to their high survivability and superior facility for degradation (Kaur et al., 2012; Moreno-Forero and van der Meer, 2015). Many *Sphingomonas* species have been determined to play important roles in plant abiotic stress tolerance, bioremediation, and biodegradation (Chen et al., 2008; Yu et al., 2013; Khan et al., 2014), as well as promoting plant growth (Yang et al., 2014; Luo et al., 2019, 2020).

Whole-genome sequencing is currently an important methodology for the discovery of useful secondary metabolites. Many secondary metabolites are natural products encoded by gene groups known as biosynthetic gene clusters (BGCs), which can be identified *via* whole-genome annotation. Various secondary metabolites including non-ribosomal peptide synthases, type I and type II polyketide synthases, lanthipeptides, lasso peptides, sactipeptides, and thiopeptides can be detected using the antiSMASH tool for genome mining (Blin et al., 2021). To survive in diverse environments, some members of the genus *Sphingomonas* are known to produce carotenoids such as lycopene, β-carotene, zeaxanthin, caloxanthin, astaxanthin, and nostoxanthin (Jenkins et al., 1979; Kawahara and Kuraishi, 1999; Zhu et al., 2012; Kim et al., 2014), whose antioxidant and anticancer properties reduce stress arising from the environments. Carotenoids are members of the isoprenoids group with diverse structures and functions, which are all synthesized *via* the common precursor isopentenyl diphosphate (IPP) or its isomer dimethylallyl diphosphate (DMAPP). The carotenoid biosynthesis pathway begins with the condensation of two molecules of geranylgeranyl pyrophosphate to produce phytoene, then converted into lycopene *via* phytofluene, ζ-carotene, and neurosporene in four desaturation steps, and finally to lycopene, then cyclization produces the various carotenes, such as β-carotene, xanthophylls such as zeaxanthin are oxygenation products of carotenes. Among these carotenoids, nostoxanthin ((2R,3R,2′R,3′R)-β or β-Carotene-2,3,2′,3′-tetrol) is a yellow pigment in the xanthophyll group of the carotenoid family having the molecular formula C_40_H_56_O_4_. It is biosynthesized from the precursor zeaxanthin. Two hydroxylations produce caloxanthin followed by nostoxanthin (Zhu et al., 2012). Nostoxanthin is a natural pigment and is found in many strains of the genus *Pseudomonas* (Jenkins et al., 1979), some *Sphingomonas* strains (Kikukawa et al., 2021), *Sphingobium* sp. (Liu et al., 2021), and cyanobacteria (Silkina et al., 2019). The marine bacteria *Erythrobacter flavus* (Setiyono et al., 2019) was also reported to produce nostoxanthin. Although one excellent review analysis of the genetic diversity of carotenogenesis in bacteria of the order Sphingmonasdales, is presumptive that some *Sphingomonas* members produce nostoxanthin based on the color of strain and presence of *crt* ORF, still a need to identify the pigment and well-describe the biosynthesis pathway and other genomic details in genus *Sphingomonas* (Siddaramappa et al., 2018). Besides, many research suggests that carotenoids such as zeaxanthin, and astaxanthin play the antioxidant role that scavenges ROS and free radicals and/or free radicals in the lipid phase of the plant cell membrane, helping the plant alleviate salt stress (Zhang et al., 2012, 2016; Ren et al., 2021; Song et al., 2022). In the present study, a new strain designated as AK-PDB1-5^T^ was isolated from the needle-like leaves of the Korean fir. The Korean fir is an endemic, rare, and valuable tree species that suffered large dieback and a severe decline for unknown reasons. As a traditional medicine plant (Yeşilada et al., 1995), the Korean fir can produce many important secondary metabolites such as essential oils (Yoon et al., 2009), lanostane terpenoids (Kim et al., 2004), and secocycloartenoids (Kim et al., 2001) that can be used to treat hypertension, uterine bleeding, human skin and lung cells (Ahn et al., 2020), and tuberculosis (Chinthanom et al., 2021). More and more researchers support that some biological properties of plant-based compounds could be due to the interaction of endophytes and plants (Zhao et al., 2011; Aly et al., 2013; Singh et al., 2021; Urumbil and Anilkumar, 2021). Endophytes provide bioactive secondary metabolites to the host. The anti-inflammatory properties of some plants are due to compounds with anti-inflammatory activity that are secreted by endophytes (Fadiji and Babalola, 2020; Urumbil and Anilkumar, 2021). In our study, we investigated endophyte-derived natural compounds from a native medicinal plant. The bacterial community of the Korean fir was studied. Eighty-one endophytic strains were isolated from surface-sterilized needle leaves of *Abies koreana*. Among all the isolates, 10 strains belonged to the genus *Sphingomonas*. Genus *Sphingomonas* with the most diverse and the second most popular that attracted us to study. Strain AK-PDB1-5^T^ was isolated and characterized as a novel species of the genus *Sphingomonas*, based on the results obtained from a polyphasic taxonomic study, and genome mining detected a zeaxanthin gene cluster tightly linked to the zeaxanthin biosynthesis pathway. Finally, based on its chemical structure and whole-genome annotation, a yellow pigment produced by this bacterium has been characterized as nostoxanthin. The nostoxanthin-producing strain AK-PDB1-5^T^ alleviates the salt stress of Arabidopsis seedlings by scavenging the reactive oxygen species (ROS).

## 2. Materials and methods

### 2.1. Bacterial strains

Strain AK-PDB1-5^T^ is an endophytic bacterium isolated from the needle-like leaves of the Korean fir (*Abies koreana* Wilson) which grows on the upper part of the Mt. Halla on Jeju island in Korea (33°21′42″N 126°31′45″E). Samples were collected and placed into sterile plastic bags. Five grams of sample were surface-sterilized with 1.05% sodium hypochlorite for 10 min, followed by rinsing 5 times in sterile distilled water. After grinding with 10 ml of PBS (phosphate buffered saline) buffer, then serially diluted from 10^−1^ to 10^−4^ with PBS buffer. An aliquot of 100 μl of diluted sample was spread on potato dextrose agar (PDA, Difco) and incubated at 25°C for 7 days. Single colonies were obtained and subsequently streaked on fresh PDA. A circular, smooth, opaque strain containing a light yellow pigment was designated as AK-PDB1-5^T^ and selected for further study. The strain was preserved in sterile skimmed milk (10%, *w*/*v*) at −80°C, and deposited in the Korean Collection for Type Cultures (KCTC) and the China Center for Type Culture Collection (CCTCC) with the respective accession numbers KCTC 82822^T^ and CCTCC AB 2021150^T^. Unless otherwise stated, the cells were grown on PDA for 4 days for subsequent tests.

### 2.2. Phenotypic and biochemical characterization

The morphological characteristics of strain AK-PDB1-5^T^ were observed using scanning electron microscopy (Quanta 250 FEG) at the KRIBB Microscopy Core Facility after the strain was grown on PDA medium for 3 days. Cellular motility was tested by growth on semi-solid PDA medium (0.4% agar). Gram reactions were determined with a Gram staining kit (Difco, Korea) following the manufacturer’s instructions. Bubble production indicated positive catalase activity after 3% (*v*/*v*) hydrogen peroxide solution was added to fresh cells (Lee and Jeon, 2017), and an oxidase reagent kit (bioMérieux) was used to determine oxidase activity. Various media were used to optimize the growth of strain AK-PDB1-5^T^, including nutrient agar (NA, beef extract 3 g/l, peptone 5 g/l, and agar 15 g/l), PDA, Luria-Bertani agar (LB), Trypticase Soy Agar (TSA), marine agar 2216 (MA), reasoner’s 2A agar (R2A), and YEP medium. The optimal temperature was determined by growing the strain at various temperatures including 4, 10, 15, 20, 25, 30, 37, 40, 45, 50, and 60°C for 7 days. NaCl tolerance and the pH range for growth were measured in PD broth (PDB), and pH values ranging from 3.0 to 12.0 were adjusted with 1 N HCl and NaOH, while tested salt concentrations encompassed 0–15% (*w*/*v*; 1% concentration increments; Lee et al., 2019). Other biochemical characteristics, such as the substrate utilization, acid production from carbohydrates, and enzyme activities were tested using API 20NE (bioMérieux) or API ZYM with NaCl 0.85% medium (bioMérieux) and API 50CH according to the manufacturer’s protocol. All closely related type strains were tested under the same conditions.

### 2.3. 16S rRNA gene sequence analysis

Amplification of the 16S rRNA gene was done using PCR with bacterial DNA as the template and the universal primers 27F (5′-AGAGTTTGATCMTGGCTCAG-3′) and 1492R (5′-TACGGYTACCTTGTTACGACTT-3′; Lane, 1991). Sequencing of the PCR products was carried out by Macrogen, Inc. (Republic of Korea) using the 27F, 1492R, 518F (5′-CCAGCAGCCGCGGTAATACG-3′), and 800R (5′-TACCAGGGTATCTAATCC-3′) primers. The Vector NTI software (1.6.1) was used for assembling the sequence results and finally, nearly a full-length 16S rRNA sequence was obtained. The GenBank^2^ and the EzBioCloud databases^3^ (Yoon et al., 2017) were used to compare 16S rRNA sequence similarity. The 16S rRNA genes of closely related strains were downloaded from EZbiocloud. After multiple alignments by clustalW, gaps at the 5′ and 3′ ends were deleted by BioEdit. A 16S rRNA gene-based phylogenetic tree was constructed using the Molecular Evolutionary Genetics Analysis (MEGA 10.0) software (Kumar et al., 2018) employing the neighbor-joining (NJ), minimum-evolution (ME), and maximum-likelihood (ML) algorithms with 1,000 bootstrap iterations and Kimura two-parameter model. *Usitatibacter rugosus* 0125-3^T^ was used as an outgroup.

### 2.4. Chemotaxonomic characterization

Chemotaxonomic features of strain AK-PDB1-5^T^, including cellular fatty acids, polar lipids, and quinones, were investigated. To determine cellular fatty acids, fresh cells were harvested from PDA medium, and the whole-cell fatty acid methyl esters (FAME) were extracted according to the instructions for the standard MIDI (Sherlock Microbial Identification System version 6.0), and then analyzed by gas chromatography (Model 6,890 N; Agilent) based on the Microbial Identification software package (Sasser, 2006). Isoprenoid quinones were extracted from 100 mg of freeze-dried cells by shaking them in a chloroform/methanol mixture (2:1, v/v), and purified by thin-layer chromatography as described by Collins et al. (1980). Finally, reverse-phase high-performance liquid chromatography with ultraviolet (UV) absorbance detection at 270 nm was carried out. Polar lipids were extracted from 100 mg of freeze-dried cells with a chloroform/methanol mixture (1:2, *v*/*v*), followed by identification by two-dimensional thin-layer chromatography on Kieselgel 60 F254 plates (silica gel, 10 × 10 cm; Merck). In addition, 0.2% ninhydrin (Sigma-Aldrich), molybdenum blue (Sigma-Aldrich), 4% phosphomolybdic acid reagent, and Dragendorff’s solution were sprayed onto the plates to detect amino group-containing lipids, sugar-containing lipids, phosphorus-containing lipids, total lipids, and quaternary nitrogen-containing lipids, respectively. Polyamines were extracted from freeze-dried cells and analyzed as described by Busse and Auling (1988).

### 2.5. Genomic sequencing and annotation

A genomic DNA purification kit (MGmed, Republic of Korea) was used to isolate the genomic DNA, and the genomic DNA quantity and quality were evaluated with the PicoGreen and Nanodrop (radio A260/A280). The whole genome was sequenced by the Macrogen facility (Macrogen, Korea) on the PacBio RSII (Pacific Biosciences, Inc.) and the Illumina sequencing platforms, followed by assembly with the SMRT Portal (version 2.3) *de novo* assembler. The assembled genome was checked for potential contamination by testing the 16S rRNA in the genome using the ContEst16S algorithm (Lee et al., 2017). Genome annotation was performed *via* the National Center for Biotechnology Information Prokaryotic Genome Annotation Pipeline (PGAP) and Rast SEED (Tatusova et al., 2016). Metabolic pathways were reconstructed using BlastKOALA, based on the Kyoto Encyclopedia of Genes and Genomes (KEGG) pathway database (Kanehisa et al., 2016). Genome mining for the presence of secondary metabolite gene clusters was performed *via* the antiSMASH tool^4^ (Blin et al., 2021). The dDDH, ANI values, and orthoANI values between AK-PDB1-5^T^ and closely related strains were determined using the Genome-to-Genome Distance Calculation (GGDC) webserver^5^ (Meier-Kolthoff et al., 2013), the ANI calculator,^6^ and the standalone Orthologous Average Nucleotide Identity (OAT) software (Lee et al., 2016), respectively. The closely related strains were obtained from NCBI GenBank websites that were publicly available, and genome quality was evaluated by the Microbial Genomes Atlas (MiGA) webserver (Rodriguez et al., 2018). A whole-genome-based phylogenomic tree was constructed using the up-to-date bacterial core gene set and pipeline (UBCG) as described by Na et al. (2018), *Usitatibacter rugosus* 0125-3^T^ was used as an outgroup.

### 2.6. Nostoxanthin structure determination

Freeze-dried cells were used for carotenoid identification. An aliquot of 100 mg of freeze-dried sample was mixed with 10 ml of methanol, then shaken at 200 rpm at 25°C for 12 h. The resultant methanol extract was centrifuged at 8,000 rpm for 10 min. The UV–Vis absorption spectra were recorded from 200 to 800 nm using a microplate spectrophotometer (Multiskan Skyhigh, Thermo Fisher Scientific). The methanol extract was filtered through a 0.2 μm membrane filter and analyzed using an HPLC system (Shimadzu Corporation, Kyoto, Japan), with a YMC carotenoid (250 × 4.6 mm. D, S-5 μm, YMC) column. The separation column temperature was 35°C, with solvent A consisting of methanol-MTBE-water (85:10:5, *v*/*v*/*v*), and solvent B of 100% MTBE, as the mobile phase. The flow rate was 1 ml/min, the wavelength was set at 450 nm, and a 20 μl sample was injected. For identification of the structure, the sample was five-fold dilution and stored at −20°C until a subsequent analysis by an ultra-performance liquid chromatography-electrospray ionization-mass spectrometry (UPLC-ESI-MS) method. HR-ESI-MS spectra were obtained using a Waters ACQUITY UPLC system (Waters Corp., Milford, MA, United States) connected to a quadrupole time of flight mass spectrometer (Xevo G2-XS QTOF, Waters Corp.) from the Korea Basic Science Institute (KBSI, Chuncheon Center).

### 2.7. Assessment of plant growth and salt stress condition

Arabidopsis (*Arabidopsis thaliana*) seeds were surface sterilized with 70% ethanol for 5 min, 2.5% sodium hypochlorite for 15 min, and rinsed three times with sterile distilled water for 5 min. All sterilized seeds were sown on half-strength Murashige & Skooog (MS) medium (MS basal 2.2 g/l, sucrose 10 g/l, 2-MES 0.5 g/l, bacto-agar 15 g/l) and plants were grown in a growth chamber at 22°C and 55–60% relative humidity (RH) with a 16-h light/8-h dark cycle. Seven-day-old seedlings were co-cultured with strain AK-PDB1-5^T^ and untreated control for 10 days.

The root fresh weight, leaf fresh weight as well as chlorophyll contents (equation: *y* = 8.02 × A_663_ + 20.2 × A_645_) were recorded to estimate the plant growth condition. Differences between untreated controls and AK-PDB1-5^T^ co-cultivation under the normal and salt condition were analyzed using a two-way ANOVA followed by Sidak’s multiple comparison test in GraphPad Prism (v.8.4.3) program.

### 2.8. Reactive oxygen species (ROS) detection

To detect the reactive oxygen species, plant roots grown on 1/2MS medium containing 0 mM and 100 mM NaCl were collected from the plates, washed with 1×PBS buffer, stained root cells by adding 5 mM DCFH-DA in 5 mM sodium phosphate buffer (pH 7.0) and incubated for 30 min. The stained root tips were observed under fluorescent microscopy (ECLIPSE 80i) with FITC filter (515 nm). The fluorescent intensities were measured by Image J. Differences were analyzed by multiple *t*-tests in GraphPad Prism (v.8.4.3) program.

## 3. Results and discussion

### 3.1. Phenotypic and biochemical characterization

Strain AK-PDB1-5^T^ was isolated from the needle-like leaves of Korean fir. It was found to grow well on PDA, R2A, and grow poorly on LB, NA, TSA, and YEP (optimum PDA and R2A), but not on MA. Strain AK-PDB1-5^T^ was observed to be Gram-negative, non-motile, and rod-shaped, without flagella, 0.3–0.5 μm in width and 0.9–1.2 μm in length (Supplementary Figures S1AB). The colonies were 2–6 mm in diameter, and creamy white to yellowish. The organism grew at 4–37°C (optimal temperature 25–30°C) in the 0–0.5% salt (optimal 0%), and at pH 6.0–9.0 (optimum pH 8.0). The strain tested oxidase- and catalase-positive. Other characteristics that distinguished strain AK-PDB1-5^T^ from other closely related strains are shown in Table 1.

**Table 1:** Differential physiological and biochemical characteristics of strain AK-PDB1-5^T^ from closely related type strains.

Characteristic	1	2	3	4
Isolation source	*Abies koreana*	Soil^a^	Clinical^b^	Lava forest^c^
Colony shape	Short-rod-shaped	Short-rod-shaped^a^	Rod-shaped^b^	Rod-shaped^c^
Oxidase	Positive	Negative^a^	Positive^b^	Positive^c^
Catalase	Positive	Negative^a^	Positive^b^	Positive^c^
Motile	Non-motile	ND^a^	Motile^b^	Non-motile^c^
Cell size (μm)	0.3–0.5	0.6–0.8	0.7×1.4^b^	0.4–0.6
	X0.9–1.2	X1.1–1.5^a^		X0.7–1.0^c^
Growth at:				
Temperature range (°C)	4–37 (25–30)	10–37 (25)^a^	5–42 (30)^b^	10–30 (25)^c^
NaCl tolerance (%, *w*/*v*)	0	1 (0)^a^	ND	0–3^c^
pH range	6.0–9.0 (8)	5–8 (6.6)^a^	ND	6.0–8.0 (6.0–6.5)^c^
Assimilation (API 20NE)				
Nitrate reduction	W	−	−	−
Esculin hydrolysis	+	W	+	W
β-galactosidase	+	−	+	−
Glucose assimilation	−	−	+	−
Arabinose	−	−	+	−
Mannose	−	−	+	−
N-acetylglucosamine	−	−	W	−
Potassium gluconate	−	−	+	−
Acid production API 50CH				
L-arabinose	+	−	−	−
D-xylose	+	−	+	−
D-galactose	+	−	+	−
D-glucose	+	−	+	−
D-fructose	−	−	+	−
L-rhamnose	+	−	−	−
Methyl-α-D-mannopyranside	−	−	+	−
Methyl-α-D-glucopyranside	−	−	+	−
N-Acetylglucosamine	−	−	+	−
Amygdalin	+	−	+	−
Arbutin	+	−	+	−
Esculin ferric citrate	+	+	+	−
Salicin	+	−	W	−
D-cellobiose	+	−	+	−
Maltose	−	−	+	−
D-lactose	−	−	+	−
D-saccharose	−	−	+	−
D-trehalose	−	−	+	−
D-melezitose	−	−	+	−
D-raffinose	−	−	+	−
D-fucose	+	−	+	−
Potassium 5-keto-gluconate	−	−	−	−
ZYM				
Lipase (C14)	−	W	+	−
Leucine arylamidase	+	+	+	+
Valine arylamidase	−	+	+	W
Cystine arylamidase	−	W	+	−
Trypsin	W	+	+	−
α-chymotrypsin	−	+	+	+
α-galactosidase	−	+	+	−
β-galactosidase	+	+	+	−
β-glucuronidase	+	+	−	W
α-glucosidase	+	+	+	+
β-glucosidase	+	+	+	−
N-acetyl-β-glucosaminidase	−	+	+	−

a Zhang et al. (2017).

b Holmes et al. (1977).

c Lee et al. (2015).

Strains: 1, *Sphingomonas nostoxanthinifaciens* AK-PDB1-5^T^; 2, *S. crusticola* KCTC 42801^T^; 3, *S. paucimobilis* KCTC 2346^T^; 4, *S. vulcanisoli* KCTC 42454^T^. All data are from the present study unless indicated otherwise. +, positive; W, weakly positive; −, negative; ND, not detected.

### 3.2. Phylogenetic analyzes

**Figure 1:**
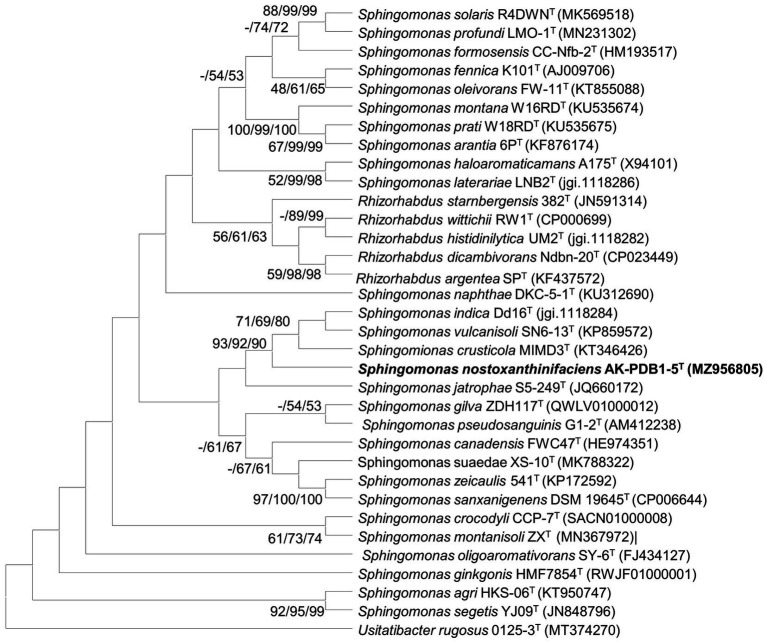
Maximum-likelihood (ML) algorithm phylogenetic tree based on 16S rRNA gene of strain AK-PDB1-5^T^. Bootstrap values (> 70%) calculated using the NJ, ML, and ME algorithms are shown.

The nearly full-length 16S rRNA gene amplicon of strain AK-PDB1-5^T^ was 1,419 nucleotides long (MZ956805). Based on the 16S rRNA gene sequence analysis, strain AK-PDB1-5^T^ appeared to belong to the genus *Sphingomonas*. A comparative analysis of the 16S rRNA sequence showed the highest similarity to *S. crusticola* MIMD3^T^ (95.6%) and *S. jatrophae* S5-249^T^ (95.3%) with values between 93.9 and 95.2% to the top 25 type strains in the genus *Sphingomonas*. To provide an ecological insight, the 16S rRNA gene sequence of strain AK-PDB1-5^T^ was compared to all the publicly available sequences on NCBI including environmental sequences and sequences from non-type strains. Results showed strain AK-PDB1-5^T^ shared a high similarity (16S rRNA gene sequence more than 97% identity) with two uncultured *Sphingomonas* isolates from environmental samples (strain Plot29-2F10, accession number: EU202874), and one uncultured bacterium from pig deep litter system (clone A702, accession number: KM456152). Based on the novel species recognition threshold of 98.6% (Kim et al., 2014), strain AK-PDB1-5^T^ was designated as a novel species in the genus *Sphingomonas*. The 16S rRNA gene sequence-based phylogenetic trees using NJ showed that strain AK-PDB1-5^T^ formed clusters with *S. crusticola* MIMD3^T^, *S. jatrophae* S5-249^T,^ and *S. vulcanisoli* SN6-13^T^. The ML and ME algorithms phylogenetic tree also supported this result (Figure 1). Therefore, *S. crusticola* MIMD3^T^ (KCTC 42801^T^) and *S. vulcanisoli* SN6-13^T^ (KCTC 42454^T^) as well as the type species *S. paucimobilis* KCTC 2346^T^ were selected as reference species for further comparative testing.

### 3.3. Chemotaxonomic characterization

The major cellular fatty acids (> 10%) of strain AK-PDB1-5^T^ and closely related strains are listed in Table 2. The most abundant fatty acids of strain AK-PDB1-5^T^ were C_14:0_ 2OH (10.6%)_,_ C_16:0_ (16.9%) and summed featured 8 (64.5%). Similar cellular fatty acid contents are consistent with closely related strains. However, the results for *S. crusticola* MIMD3^T^ (KCTC 42801^T^) also include C_19:0_ cyclo ω8*c*, while the cellular fatty acid profile of *S. vulcanisoli* SN6-13^T^ (KCTC 42454^T^) does not contain C_16:0_, and *S. paucimobilis* KCTC 2346^T^ does not contain C_14:0_ 2OH_,_ which can differentiate strain AK-PDB1-5^T^ from the most closely related strains.

**Table 2:** Cellular fatty acid profiles (> 1%) of strain AK-PDB1-5^T^ and closely related species.

Fatty acid	1	2	3	4
C_14:0_	0.4	ND	1.5	0.5
C_16:0_	**16.9**	**15.9**	9.6	**17.1**
C_16:1_ ω5c	0.9	ND	0.7	0.8
C_17:0_	ND	ND	0.7	0.7
C_17:1_ ω6c	ND	4.7	2.9	5.8
C_19:0_ cyclo ω8c	0.5	**14.8**	ND	0.5
C_18:0_	0.5	0.5	0.9	ND
C_18:1_ ω5c	1.0	1.0	3.6	ND
C_8:0_ 3OH	0.5	ND	ND	ND
C_12:0_ 2OH	2.0	ND	ND	4.0
C_13:0_ 2OH	ND	ND	ND	0.6
C_14:0_ 2OH	**10.6**	**14.5**	**10.2**	6.7
C_15:0_ 2OH	ND	1.4	0.6	ND
^*^Summed feature 3	2.4	ND	1.4	4.4
^*^Summed feature 8	**64.5**	**47.3**	**68.0**	**59.0**

^*^Summed features are groups of two or three fatty acids that cannot be separated by GLC with the MIDI System. Summed feature 3 contains C_16:1_ ω6c or/and C_16:1_ ω7c. Summed feature 8 was listed as C_18:1_ ω7c or/and C_18:1_ ω6c. Values are percentages of total fatty acids. ND, not detected. The major components (> 10%) are shown in bold. Strains: 1, *Sphingomonas nostoxanthinifaciens* AK-PDB1-5^T^; 2, *S. crusticola* KCTC 42801^T^; 3, *S. paucimobilis* KCTC 2346^T^; 4, *S. vulcanisoli* KCTC 42454^T^. All the data were obtained in the present study.

Consistent with the genus *Sphingomonas*, Q-10 was found as the predominant isoprenoid of strain AK-PDB1-5^T^, and the polar lipid profile consisted of a mixture of phosphatidylethanolamine (PE), sphingoglycolipid (SGL), phosphatidylglycerol (PG), phospholipids (PL), and lipids (L; Supplementary Figure S2). The polar lipid profile of strain AK-PDB1-5^T^ was similar to those of profiles identified from the closely related strains (Supplementary Figure S2). Although the enzyme for PC biosynthesis was annotated from the whole genome, PC was not detected in the polar lipids test. The polyamine patterns detected in strain AK-PDB1-5^T^ were for homospermidine (53.4%), putrescine (41.0%), and spermidine (15.6%; Supplementary Figure S3). The major polyamines were consistent with those reported for members of the genus *Sphingomonas* (Zhang et al., 2010; Margesin et al., 2012; Sheu et al., 2015).

### 3.4. Genome properties and genetic relatedness

**Figure 2:**
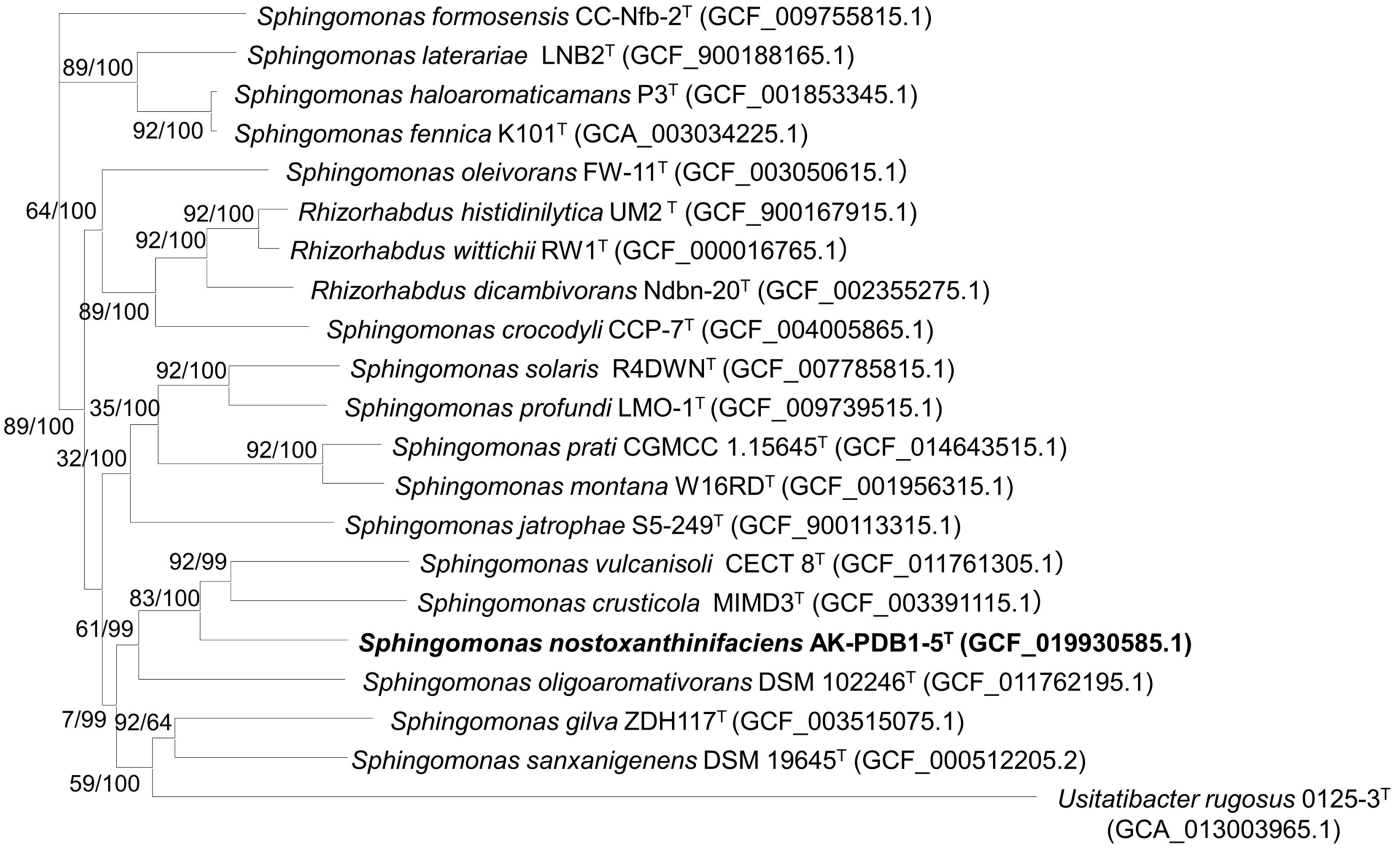
Maximum-likelihood (ML) algorithm phylogenomic tree based on up-to-date bacterial core genes (UBCGs; concatenated alignment of 92 core genes) showing the relationship between strain AK-PDB1-5^T^ and other members of the genus *Sphingomonas*. Gene support index (GSI, left) and bootstrap values (right) are indicated at the nodes. Scale bar, 0.050 substitutions per position. The 92 bacterial core genes were alaS, argS, aspS, cgtA, coaE, cysS, dnaA, dnaG, dnaX, engA, ffh, fmt, frr, ftsY, gmk, hisS, ileS, infB, infC, ksgA, lepA, leuS, ligA, nusA, nusG, pgk, pheS, pheT, prfA, pyrG, recA, rbfA, rnc, rplA, rplB, rplC, rplD, rplE, rplF, rplI, rplJ, rplK, rplL, rplM, rplN, rplO, rplP, rplQ, rplR, rplS, rplT, rplU, rplV, rplW, rplX, rpmA, rpmC, rpmI, rpoA, rpoB, rpoC, rpsB, rpsC, rpsD, rpsE, rpsF, rpsG, rpsH, rpsI, rpsJ, rpsK, rpsL, rpsM, rpsO, rpsP, rpsQ, rpsR, rpsS, rpsT, secA, secG, secY, serS, smpB, tig, tilS, truB, tsaD, tsf, uvrB, ybeY, and ychF.

The whole genome of strain AK-PDB1-5^T^ comprised a single circular chromosome of 4,298,284 bp in size after assembly by the SMRT Portal *de novo* assembler (version 2.3). A comparison of two copies of 16S rRNA gene fragments from the whole-genome sequence indicated that no contaminating DNA sequences occurred during genome assembly. The G + C content calculated from the genome of AK-PDB1-5^T^ was 67.0%, similar to other *Sphingomonas* species, which had high G + C contents as indicated in a previous report for the genus *Sphingomonas* (57.4–70.5 mol%).^7^ The whole genome was deposited in NCBI under the accession number CP082839.1. A total of 3,994 protein-coding genes and 60 RNA genes, including two 5S rRNA genes, two 16S rRNA genes, two 23S rRNA genes, four ncRNAs, and 50 tRNA genes were annotated by PGAP (Supplementary Table S1). According to the cluster orthologous group (COG) annotation results, most CDSs were classified as unknown (29.5% of total assigned COGs), and general function prediction only (8.2% of the total assigned COGs), amino acid transport, and metabolism (6.0% of the total assigned COGs; Supplementary Figures S4, S5). The genome comparison between strain AK-PDB1-5^T^ and the closest species showed ANI values in a range of 75.1–76.8%. The dDDH values ranged from 19.5 to 21%, and the OrthoANI values ranged from 73.8 to 76.5 (Supplementary Figure S6), which also supported that the strain was a novel species in the genus *Sphingomonas*, considering that the values obtained were significantly lower than the proposed dDDH (< 70%) and ANI cutoff (95–96%) values for bacterial species delineation (Chun et al., 2018). A circular map of the stain AK-PDB1-5^T^ is shown in Supplementary Figure S2. The whole genome of closely related strains obtained from NCBI GenBank showed a relatively high quality (more than 83.2) by employing MiGA webserver to assess the quality/completeness/contamination of the genome assembly (Supplementary Table S1). The whole-genome phylogenomic tree based on 92 core genes also supported that strain AK-PDB1-5^T^ formed a phylogenetic lineage within the genus *Sphingomonas*, consistent with the 16S rRNA-based phylogenomic tree (Figure 2).

Based on data obtained by the phenotypic, physiological, phylogenomic, and biochemical results support that strain AK-PDB1-5^T^ is a member of the genus *Sphingomonas*. However, a low 16S rRNA similarity (below 95.6%), ANI values (less than 75.1%), as well as low dDDH values (less than 19.5%), leads to the conclusion that strain AK-PDB1-5^T^ differs from the most closely related strains. This conclusion was further supported by the phylogenetic trees based on the 16S rRNA and core gene sets from the whole-genome sequence. Taken together, this data suggests that AK-PDB1-5^T^ is a new member of the genus *Sphingomonas*.

### 3.5. Genome-derived features of strain AK-PDB1-5^T^

Genome annotation was carried out using the RAST server and the BlastKOALA pipeline to reconstruct the metabolic pathways. The following sections are predicted from the genome sequences (Supplementary Figure S7).

#### 3.5.1. Phenotypic and biochemical features

##### 3.5.1.1. Motility

Genes related to flagellar motility in prokaryota were detected in the whole genome, such as the flagellar motor switch protein FliM, the basal-body rod protein FlgC, the flagellin protein FlaA, the flagellar L-ring protein FlgH, the flagellar motor switch protein FliN, the flagellar biosynthesis proteins FlhB, FlhA, FliR, the flagellar motor rotation proteins MotA, MotB, the flagellum specific ATP synthase FliI, and the flagella basal-body rod modification protein FlgD. More than 11,000 amino acids in the genus *Lactobacillus* (Cousin et al., 2015), and 27 proteins in the genus *Flavobacteriaceae* were related to flagella motility (Gavriilidou et al., 2020). The gene cluster of flagellar motility for *Sphingomonas* is poorly characterized. Although many flagellar assembly genes were annotated in the genome of strain AK-PDB1-5^T^, it may absence of some genetic elements since it lacks flagella and non-motile.

##### 3.5.1.2. Respiration

Genome annotation indicated the presence of all the subcategory genes related to terminal electron-accepting reactions and electron-donating reactions. Some oxidase subunits such as terminal cytochrome O ubiquinol oxidase, terminal cytochrome C ubiquinol oxidase, terminal cytochrome oxidase, and anaerobic respiratory reductases such as vanillate O-demethylase oxidoreductase (EC 1.14.13), flavodoxin reductases (ferredoxin-NADPH reductases) family 1, and ferredoxin reductase were annotated from the whole genome. The electron-donating reactions included respiratory complex I, NADH ubiquinone oxidoreductase (chain A-N), and others.

Stress response: Genes related to stress response including oxidative stress, detoxification, and periplasmic stress were detected. Osmotic stress-related genes coding for the outer membrane protein A precursor, the synthesis of osmoregulated periplasmic glucans, protection from reactive oxygen species, oxidative stress, biosynthesis, and gamma-glutamyl cycle as well as glutaredoxins were seen. Detoxification-related genes included a glutathione-dependent pathway for formaldehyde detoxification. Periplasmic stress-related genes containing the periplasmic stress response were also noted.

Polar lipid metabolism: phosphatidylethanolamine (PE), Phosphatidylcholine (PC) which is biosynthesized from PE, and sphingolipid (SGL) biosynthesis pathway were reconstructed from the whole genome *via* the BlastKOALA pipeline. The PC biosynthesis enzymes phosphatidylethanolamine/phosphatidyl-N-methylethanolamine N-methyltransferase [EC:2.1.1.17 2.1.1.71], and the PE biosynthesis enzymes phosphatidate cytidylyltransferase [EC:2.7.7.41]; CDP-diacylglycerol-serine O-phosphatidyltransferase [EC:2.7.8.8]; phosphatidylserine decarboxylase [EC:4.1.1.65] were annotated in the genome. However, a polar lipid pattern consisting of phosphatidylethanolamine (PE), sphingoglycolipid (SGL), phosphatidylglycerol (PG), phospholipids (PL), and lipid (L) was detected for strain AK-PDB1-5^T^ in two-dimensional TLC.

#### 3.5.2. Metabolite biosynthesis gene clusters analysis

Although the genus *Sphingomonas* contains 146 published species, only limited secondary metabolites were found (Mnich et al., 2017), and it remains untapped for potential applications for those natural products. AntiSMASH is an important tool for the mining of gene clusters of natural products. BGCs encode enzymes related to the secondary metabolite biosynthesis pathway. The whole genome of strain AK-PDB1-5^T^ was used to predict the secondary metabolite gene clusters by the online tool antiSMASH (Blin et al., 2021). Six BGCs regions were identified from the whole genome of AK-PDB1-5^T^ (Supplementary Table S2 and Supplementary Figures S8–S13), which contained sphingan polysaccharide, malleobactin A/malleobactin B/malleobactin C/malleobactin D and zeaxanthin. Sphingan polysaccharide is a very common compound in this genus, exemplified by compounds such as WL gum, gallen gum, rhamsan gum, and welan gum (Zheng et al., 2018; Baez et al., 2019; Li et al., 2019; Zhu et al., 2019 2020, 2021; Huang et al., 2020; Liu et al., 2020; Xu et al., 2020; Ke et al., 2021; Zhao et al., 2021). The sphingans group has applications in a variety of industries such as food, cement, personal care, abiotic stress tolerance, bioremediation, and biodegradation, etc. Malleobactin is a member of the siderophore family and has an important role in plant resistance to pathogens (Xiao et al., 2020; Saboe et al., 2021). Zeaxanthin has significant commercial value for its antioxidant potential and is widely used in the food, cosmetic and pharmaceutical industries. Some species in the genus *Sphingomonas* have been reported to produce zeaxanthin (Asker et al., 2007; Thawornwiriyanun et al., 2012); however, the biosynthesis pathway and related gene clusters are not clear. We provide the BGCs information and valuable insights into those natural products.

#### 3.5.3. Identification of the carotenoid pigment

Strain AK-PDB1-5^T^ grown on PDA medium was white-yellow in color and showed carotenoid accumulation. The genome mining tool antiSMASH was used to predict the secondary metabolite gene clusters of carotenoids, and the results suggested that the yellow color produced by strain AK-PDB1-5^T^ could be zeaxanthin, caloxanthin (zeaxanthin with one hydroxyl group added), or nostoxanthin (zeaxanthin with two hydroxyl groups added).

We analyzed a methanol extract using HPLC and some carotenoid standards including zeaxanthin, lutein, cryptoxanthin, β-carotene, α-carotene, violaxanthin, and neoxanthin were tested by HPLC at the same time. The results showed the pigment from strain AK-PDB1-5^T^ had a retention time of 4.5 min and a minor peak with a retention time of 5.5 min (Supplementary Figure S14). However, these peaks did not correspond to the peaks produced by authentic standards of zeaxanthin, lutein, cryptoxanthin, β-carotene, α-carotene, violaxanthin, or neoxanthin (Supplementary Figure S14). Compared to zeaxanthin, the main peak of AK-PDB1-5^T^ had a shorter retention time, because the carotenoid from strain AK-PDB1-5^T^ has higher hydrophilicity than zeaxanthin.

**Figure 3:**
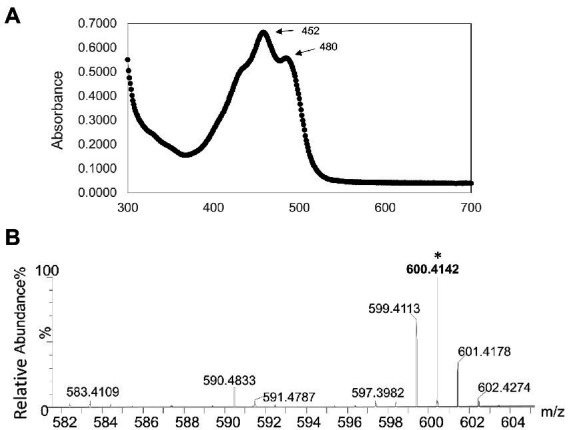
Identification of the carotenoid produced by strain AK-PDB1-5^T^ **(A)** Ultraviolet-visible absorption spectra (UV–Vis) of the major carotenoid from AK-PDB1-5^T^ **(B)** ESI-MS ion spectra of the carotenoids from AK-PDB1-5^T^.

UV–Vis absorption spectroscopy was performed to determine the absorption spectra of a methanol extract from strain AK-PDB1-5^T^, and showed highly absorbent peaks at 452 and 480 nm (Figure 3A). A previous study showed that β-carotene produced a strong band with subpeaks at 481, 454, and near 425 nm. On the other hand, zeaxanthin and caloxanthin, a hydroxyl-substituted derivative of β-carotene, has three bands with a shape almost identical to those of β-carotene (λmax values at 422, 450, and 476 nm; Zhu et al., 2012; Takaya et al., 2018), canthaxanthin (473 nm), nostoxanthin (λmax at 427, 452, and 480 nm; Kikukawa et al., 2021), and astaxanthin (477 nm) all carbonyl-substituted derivatives of β-carotene show absorption bands much less structured than those of β-carotene (Takaya et al., 2018). The strain AK-PDB1-5^T^ spectra were highly matched with nostoxanthin (λmax values at 452 and 480 nm; Takaya et al., 2018) and close to that of zeaxanthin (λmax values at 422, 450, and 476 nm; Pavelková et al., 2020).

To identify the carotenoid produced by strain AK-PDB1-5^T^, an analysis was conducted by UPLC equipped with an electro-spray ionization (ESI) time-of-flight (TOF) mass spectrometer (MS), and MS/MS systems. The ESI-MS profile of all the extracts was found to be comparable with the standard, the zeaxanthin MS/MS spectra data of [M + H]^+^: 569, caloxanthin MS/MS spectra data of [M + H]^+^: 569, β-carotene MS/MS spectra data of [M + H]^+^: 537, while nostoxanthin spectra data of m/z 600.4243 [M + H]^+^ (Zhu et al., 2012). The molecular formula of the carotenoid from strain AK-PDB1-5^T^ was determined to be C_40_H_56_O_4_, with ESI MS spectra data of m/z 600.4243 [M + H]^+^ and m/z 583.4109 [M + H-18]^+^ (Figure 3B). The ion patterns produced were same as the MassBank Record: CA000155 that is available online Nostoxanthin Mass Spectrum (massbank.jp) and as those from previous studies (Zhu et al., 2012). Based on the results above, the carotenoid produced by strain AK-PDB1-5^T^ was identified as nostoxanthin.

#### 3.5.4. Nostoxanthin biosynthetic pathway

Nostoxanthin ((2R,3R,2′R,3′R)-β, β-Carotene-2,3,2′,3′-tetrol) is biosynthesized from zeaxanthin. Zeaxanthin, the precursor of nostoxanthin, is hydroxylzed twice, first to caloxanthin and finally to nostoxanthin (Zhu et al., 2012). Although many different carotenoid (*crt*) synthetic enzymes have been cloned from bacteria, plants and algae, much further study is still necessary to fully elucidate the biosynthesis of carotenoids as well as modifications of the final structures. To better understand the biosynthesis pathway in strain AK-PDB1-5^T^, the whole genome was used to predict it. Two separate pathways exist for the biosynthesis of IPP, including the mevalonate (MVA) pathway and the 2-C-Methyl-D-erythritol 4-phosphate (MEP) pathway, both of which are carotenoid precursors. Genome annotation analysis *via* PGAP, RAST SEED, and AntiSMASH annotations revealed that IPP from strain AK-PDB1-5^T^ is only generated *via* the MEP pathway. Some genes for some enzymes in the MEP pathway were predicted; one copy of 1-deoxy-D-xylose 5-phosphate synthase (*dxs*), one copy of 1-deoxy-D-xylose 5-phosphate reductoisomerase (*dxr*), putative intracellular septation protein A (*ispZ*), two copies of 4-hydroxy-3-methylbut-2-enyl diphosphate (*ispH*), one copy of flavodoxin-dependent(E)-4-hydroxy-3-methylbut-2-enyl-diphosphate synthase (*ispG*), one copy of phosphoenolpyruvate carboxykinase (*pck*), one copy of phosphoenolpyruvate carboxylase (*ppc*), and one copy of pyruvate kinase (*pykFA*), all for the MEP pathway. No genes for enzymes in the MVA pathway were predicted. The absence of genes for the MVA pathway reinforces the conclusion that in strain AK-PDB1-5^T^, IPP is biosynthesized *via* the MEP pathway. These results were consistent with a previous study that indicated that the MEP pathway is typically found in most bacteria and on plant plastids, while the MVA pathway is mostly present in plants and archaea (Li et al., 2020).

**Figure 4:**
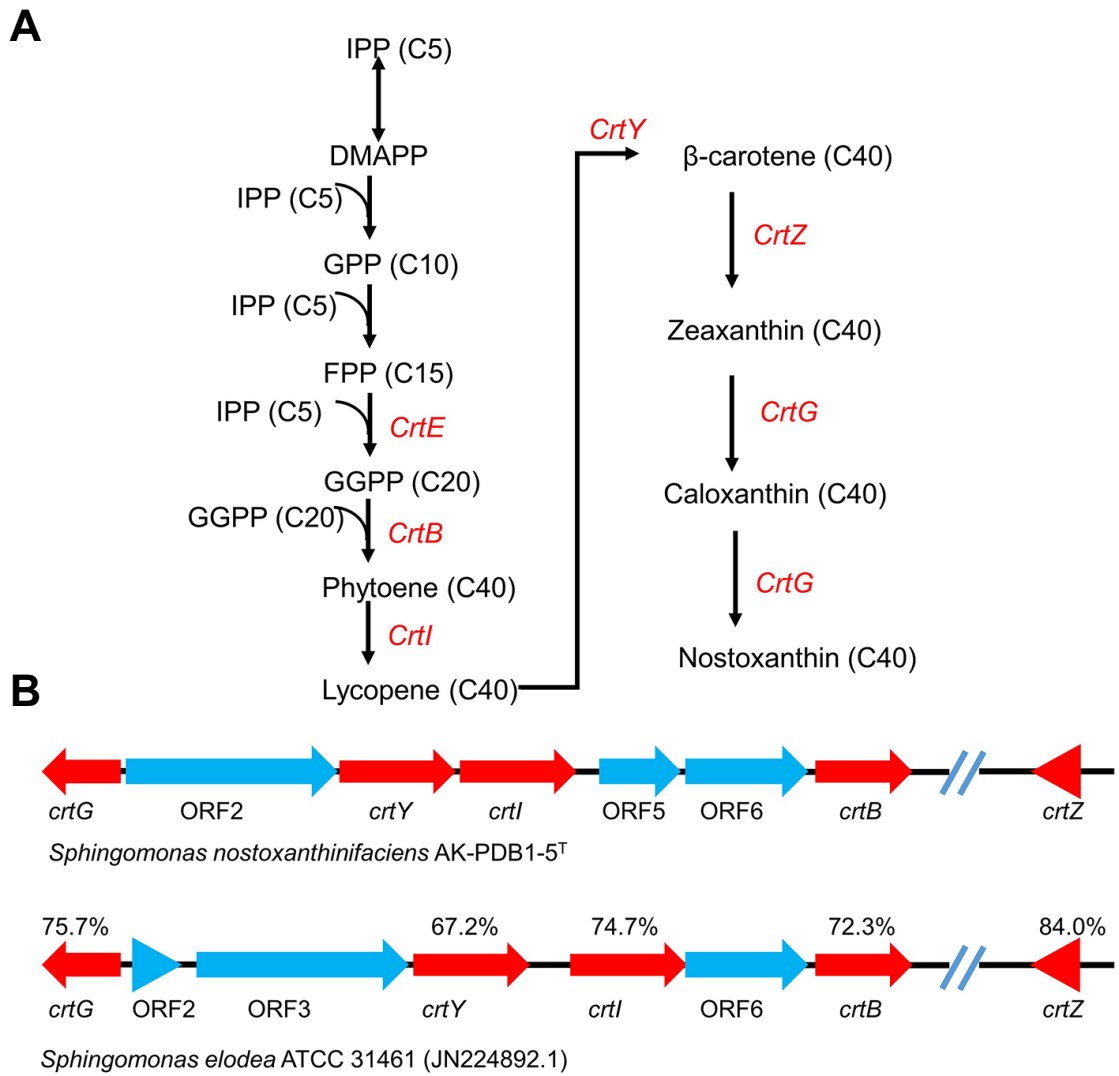
Carotenogenic gene clusters and the proposed biosynthetic pathway of nostoxanthin in strain AK-PDB1-5^T^. **(A)** comparison of the gene cluster in strain AK-PDB1-5^T^ (up) and *“Sphingomonas elodea”* ATCC 31461 (JN224892.1; bottom). The genes are presented as arrows pointing in the direction of transcription; the value below each gene designates the similarity of nucleotides between the AK-PDB1-5^T^ nostoxanthin biosynthetic enzyme and the corresponding gene from the *S. elodea* ATCC 31461. **(B)** nostoxanthin biosynthetic pathway in strain AK-PDB1-5^T^. IPP, isopentenyl pyrophosphate; DMAPP, dimethylallyl pyrophosphate; FPP, farnesyl diphosphate; GGPP, geranylgeranyl diphosphate. *crtE*, GGPP synthase; *crtB*, phytoene synthase; *crtG,* carotenoid 2,2-β-hydroxylase; *crtI*, phytoene desaturase/dehydrogenase; *crtY*, lycopene cyclase; *crtZ*, and β-carotene hydroxylase.

After IPP has been synthesized, carotenoid biosynthesis continues by growing the polyprenyl pyrophosphate chain. IPP is isomerized to geranyl PP (GPP, C10), farnesyl PP (FPP, C15), or geranylgeranyl PP (GGPP, C20) by *ispA* and *crtE* or *gps*, which are precursors of mono-, di-, and tri-terpenes and carotenoids. Next, two GGPP molecules are condensed head-to-head by phytoene synthase, resulting in the formation of the first carotene, phytoene (C40) by *crtB* (Figure 4A). Four double bonds are introduced into phytoene to form lycopene, which then becomes a precursor to a variety of carotenoids, e.g., lycopene (*crtI*), *β*-carotene (*crtL*), zeaxanthin (*crtZ*), and astaxanthin (*crtW*; Kim et al., 2014; Lee et al., 2018). Zeaxanthin is converted to the yellow pigment nostoxanthin by the gene product of *crtG*. The *crtG* gene encodes 2,2-β-hydroxylase which is responsible for hydroxylation of β-carotene (Zhu et al., 2012). In comparison with the carotenoid biosynthesis gene cluster of “*Sphingomonas elodea”* ATCC 31461 (JN224892.1), which has been reported to produce nostoxanthin, strain AK-PDB1-5^T^ showed 71.1% gene similarity to *S. elodea* ATCC 31461. The carotenoid biosynthesis gene cluster as well as nostoxanthin biosynthesis pathway of stain AK-PDB1-5^T^ has been proposed (Figure 4). PGAP, RAST SEED and AntiSMASH annotations suggest that the strain AK-PDB1-5^T^ analyzed in this study possesses the gene cluster *crtB*, *crtG*, *crtI*, *crtY*, *crtZ* to produce the carotenoid nostoxanthin from the precursor GGPP (Figure 4B). Although we did not directly find *crtG* from the whole genome, by blasting the *crtG* gene sequence of “*Sphingomonas elodea*” ATCC 31461 (JN224892.1; Zhu et al., 2012), a 76.3% gene similarity to *crtG* of “*Sphingomonas elodea*” ATCC 31461 was found in the whole genome of AK-PDB1-5^T^. Based on the annotation results from the whole genome, strain AK-PDB1-5^T^ was confirmed to produce nostoxanthin.

### 3.6. Effect of strain AK-PDB1-5^T^ on plant growth under salt condition

**Figure 5:**
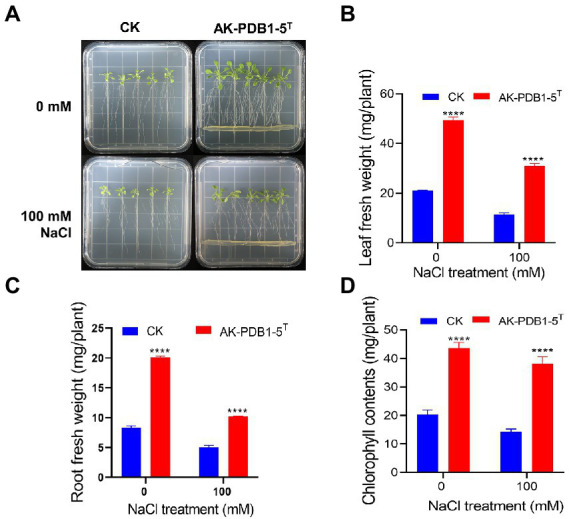
Plant growth-promoting effects of Arabidopsis seedling by strain AK-PDB1-5^T^ under normal and salt stress conditions. **(A)** Comparison of *Arabidopsis* seedling growth by control (no treatment, CK) and AK-PDB1-5^T^ co-cultivated at 22°C for 10 days under normal and salt stress conditions (100 mM NaCl). **(B–D)** Quantification of leaf fresh weight **(B)**, root fresh weight **(C)**, and chlorophyll contents **(D)** Error bars represent the standard error. Asterisks indicate significant differences between the control and AK-PDB1-5^T^ treatment under salt stress (100 mM NaCl; ^****^p < 0.0001).

To evaluate the effect of strain AK-PDB1-5^T^ on plant growth under salt conditions, 7-day-old Arabidopsis seedlings were co-cultivated with strain AK-PDB1-5^T^ under 0 mM and 100 mM of NaCl. The leaf fresh weight and root fresh weight were examined after 10 days of co-cultivation (Figure 5). The results showed strain AK-PDB1-5^T^ significantly improved the Arabidopsis seedling biomass under 0 mM and 100 mM of NaCl. Shoot fresh weight was increased 1.69-fold under salt condition (100 mM NaCl), which was increased higher compared to normal conditions (1.34-fold), while root fresh weight was increased 1.02-fold under salt condition. A similar result was found in chlorophyll content. These results showed that strain AK-PDB1-5^T^ has not only plant growth-promoting activity under normal condition, but also enhances salt tolerance when plants are exposed to salt stress.

**Figure 6:**
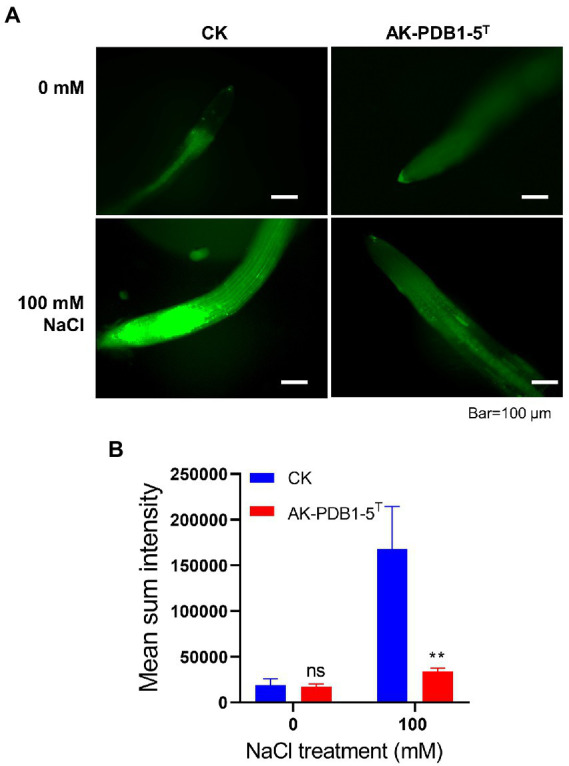
DCFH-DA staining of ROS in roots. **(A)** DCFH-DA straining of root tips after co-inoculation with strain AK-PDB1-5^T^ upon different salt conditions (0 and 100 mM NaCl). Scale bar: 100 μm. **(B)** The fluorescence intensities of roots were measured by ImageJ. Error bars represent the standard error. Asterisks indicate significant differences between the control and AK-PDB1-5^T^ treatment under salt stress condition (100 mM NaCl, ^**^p < 0.01).

Furthermore, we examined ROS production in *Arabidopsis* root tips under salt stress condition (Figure 6). We observed that ROS levels in roots were significantly increased under salt stress condition (100 mM NaCl; Figure 6), but the fluorescence intensity of ROS in root tips was greatly decreased by strain AK-PDB1-5^T^ (Figure 6). These results suggest that strain AK-PDB1-5^T^ alleviates salt stress in *Arabidopsis* roots by scavenging the ROS. In the previous study, it has well studied that carotenoid is known lipid protectants and active oxygen scavengers, and play an important role in antioxidant caused by ROS, these carotenoid can scavenge ROS and free radicals and/or free radicals in the lipid phase of the plant cell membrane, help the plant alleviate salt stress (Zhang et al., 2012, 2016; Ren et al., 2021; Song et al., 2022). For instance, pretreated with carotenoids (β-carotene) improved the *Lepidium sativum* growth under salt stress, as β-carotene increased the antioxidant activity of the plant and mitigated salt stress (Babaei et al., 2022); exogenous zeaxanthin alleviates pepper plant grown under low temperature and low light by reduced ROS accumulation in pepper seedlings. In the present study, nostoxanthin, as one type of yellow carotenoid, biosynthesized from zeaxanthin by the addition of two hydroxyl groups, was produced by strain AK-PDB1-5^T^, was found to reduce ROS accumulation caused by salt stress, promoted plant growth under salt stress. Except for nostoxanthin, we try to find some specific metabolic pathways that may reduce the abundance of ROS, such as a complete trehalose biosynthesis pathway, including *glgB* gene (1,4-alpha-glucan branching enzyme [EC:2.4.1.18]), *glgA* gene (starch synthase [EC:2.4.1.21]), *glgC* (glucose-1-phosphate adenylyltransferase [EC:2.7.7.27]), *treX* (isoamylase [EC:3.2.1.68]), *glgZ* (maltooligosyltrehalose trehalohydrolase [EC:3.2.1.141]), and *glgY* ((1- > 4)-alpha-D-glucan 1-alpha-D-glucosylmutase [EC:5.4.99.15]). Trehalose as a key organic osmolyte was proven to be effectively involved in plant abiotic stress. The plant enhanced tolerance to cold, salinity, and drought tolerance by exogenous trehalose. However, more studies need to be performed to understand the mechanism.

#### 3.6.1. Description of *Sphingomonas nostoxanthinifaciens* sp. nov

*Sphingomonas nostoxanthinifaciens* (nos.to.xan.thi.ni.*fa’ci.ens*. N.L. neut. n. *nostoxanthinum*, nostoxanthin; L. pres. part. faciens, making/producing; N.L. part. Adj. *nostoxanthinifaciens*, nostoxanthin-producing).

Colonies are white-yellow pigmented, circular, smooth, and opaque (2–6 mm in diameter) after growth on PDA medium at 25°C for 3 days. Strain AK-PDB1-5^T^ is found to grow well on PDA, R2A, can grow poorly on LB, NA, TSA, and YEP (optimum PDA and R2A), but not on MA. Cells are Gram-negative, catalase-, oxidase-positive, non-motile, and rod-shaped (0.3–0.5 μm in width and 0.9–1.2 μm in length), without flagella. Optimal growth occurs at 25–30°C, pH 8.0, and in 0% (w/v) NaCl.

In the API ZYM test, positive reactions for alkaline phosphate, esterase (C4), esterase lipase (C8), leucine arylamidase, acid phosphate, naphthol-AS-BI-phosphohydrolase, *β*-galactosidase, *β*-glucuronidase, *α*-glucosidase, and *β*-glucosidase, while the other substrates were negative. On API 20NE strips, esculin hydrolysis, nitrate reduction, and *β*-galactosidase showed positive reactions, while the other reactions are negative. On API 50CH strips, L-arabinose, D-galactose, D-glucose, L-rhamnose, amygdalin, arbutin, esculin ferric citrate, salicin, D-cellobiose, and D-fucose show positive reactions, and other substrates are negative. The major fatty acids are C_14:0_ 2OH_,_ C_16:0_, and summed featured 8. The respiratory quinone detected in strain AK-PDB1-5^T^ was Q-10, while sphingoglycolipid and phosphatidylethanolamine are found as the major polar lipids.

The type strain is *Sphingomonas nostoxanthinifaciens* AK-PDB1-5^T^ (= KCTC 82822^T^ = CCTCC AB 2021150^T^), isolated from needle-like leaves of the Korean fir taken from Mt. Halla, Jeju island, South Korea. The accession numbers for 16S rRNA gene and the whole-genome sequences of strain AK-PDB1-5^T^ are MZ956805.1 and CP082839.1.

## Data availability statement

The datasets presented in this study can be found in online repositories. The names of the repository/repositories and accession number(s) can be found in the article/Supplementary material.

## Author contributions

JL designed the research and supervised the project. LJ and JS carried out the experiment. LJ analyzed the results and wrote the manuscript. YP, DJ, JHL, and CK edited the manuscript. JL and LJ modified the manuscript. All authors contributed to the article and approved the submitted version.

## Funding

This work was supported by the Korea Research Institute of Bioscience and Biotechnology (KRIBB) Research Initiative Program (KGM5282223) and the Basic Science Research Program through the National Research Foundation of Korea (NRF) funded by the Ministry of Education (NRF-2020R111A2072308).

## Conflict of interest

The authors declare that the research was conducted in the absence of any commercial or financial relationships that could be construed as a potential conflict of interest.

## Publisher’s note

All claims expressed in this article are solely those of the authors and do not necessarily represent those of their affiliated organizations, or those of the publisher, the editors and the reviewers. Any product that may be evaluated in this article, or claim that may be made by its manufacturer, is not guaranteed or endorsed by the publisher.

## Footnotes

^1^ https://lpsn.dsmz.de/genus/sphingomonas

^2^ https://blast.ncbi.nlm.nih.gov/Blast.cgi

^3^ http://www.ezbiocloud.net

^4^ http://antismash.secondarymetabolites.org

^5^ http://ggdc.dsmz.de/distcalc2.php

^6^ https://www.ezbiocloud.net/tools/ani

^7^ https://www.ncbi.nlm.nih.gov/genome/?term=sphingomonas
