# Supplementary material for: A nostoxanthin-producing bacterium, Sphingomonas nostoxanthinifaciens sp. nov., alleviates the salt stress of Arabidopsis seedlings by scavenging of reactive oxygen species
Source: Front Microbiol. 2023 Feb 10;14:1101150. doi: 10.3389/fmicb.2023.1101150 (PMC9950776; doi:10.3389/fmicb.2023.1101150)
Supplement: Supplementary file 1 [file Data_Sheet_1.pdf]

Supplementary Information

A

B

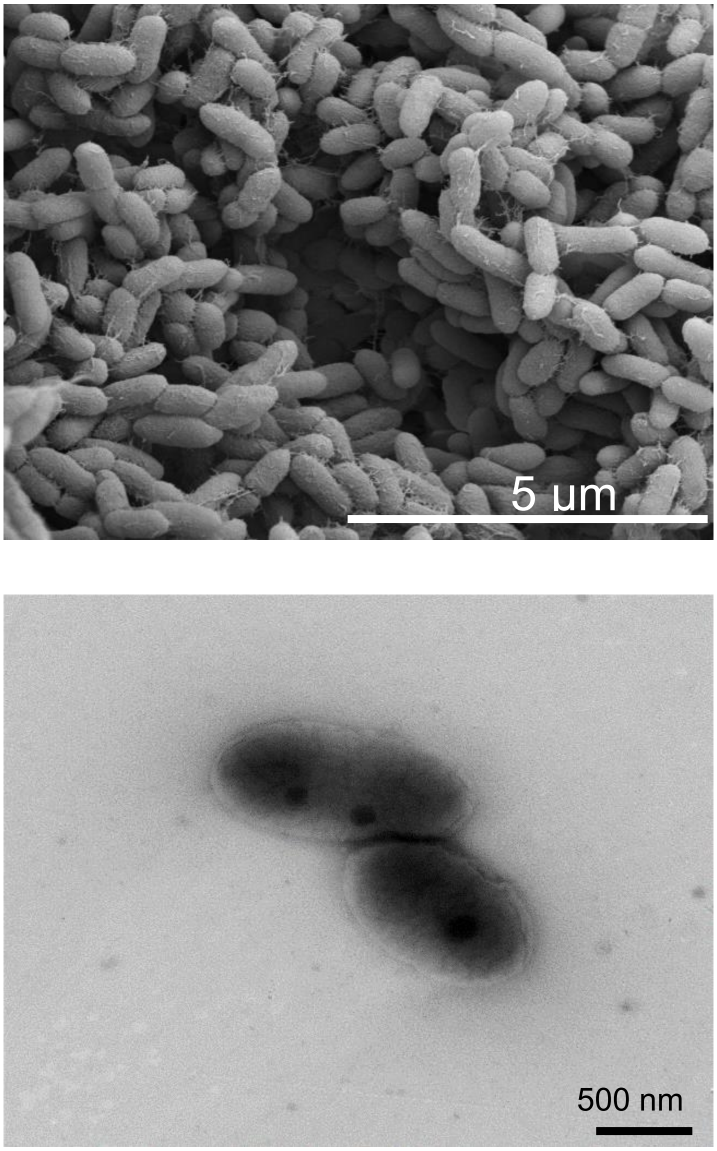

Figure S1. EM images of strain AK-PDB1-5^{T} . A, Scanning electron micrograph of strain AK-PDB1-5^{T}. Scale bar = 5 µm. B, Transmission electron microscopy of strain AK-PDB1-5^{T} **.** Scale bar=500 nm.

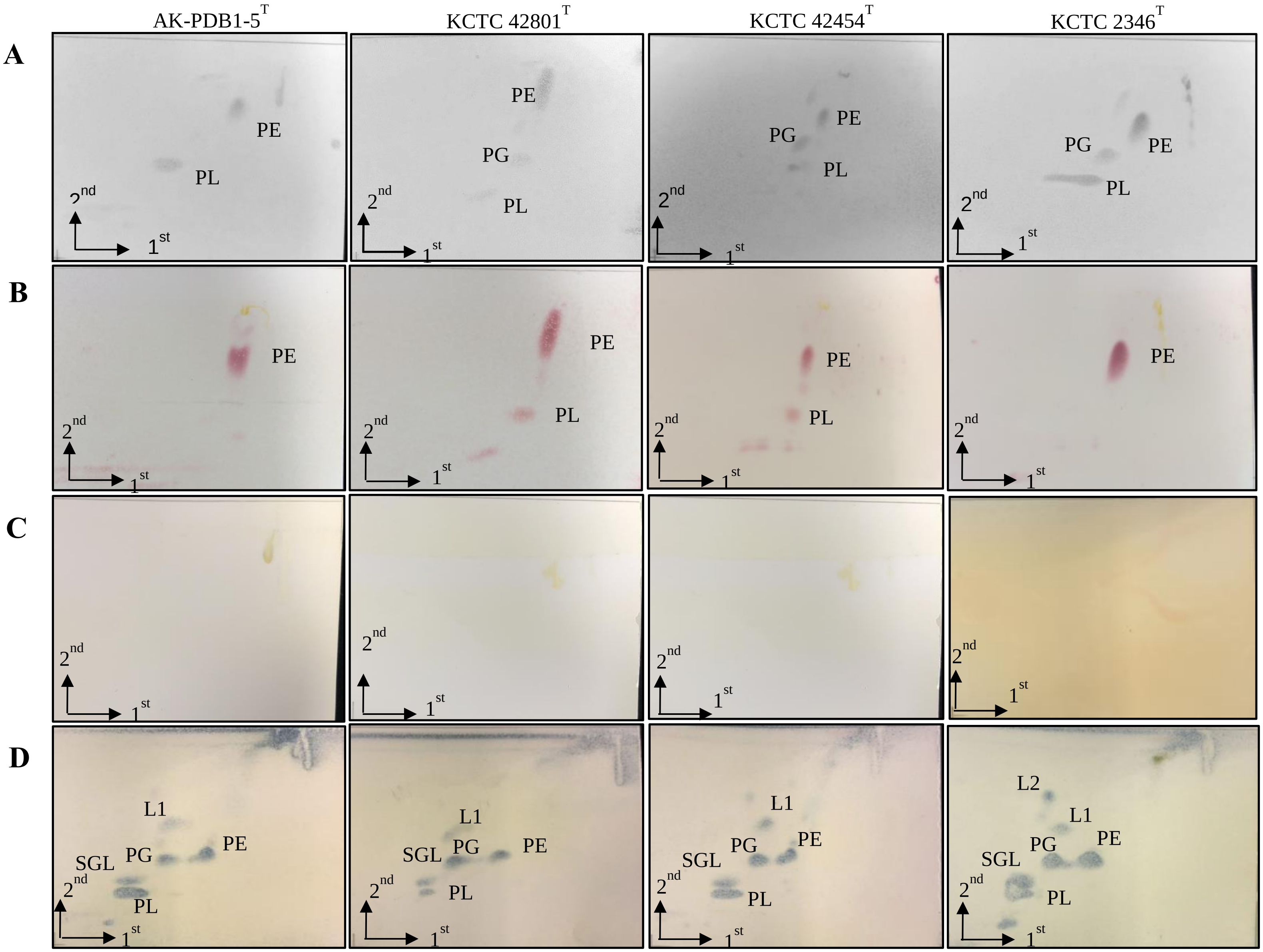

AK-PDB1-5T
KCTC 42801T
KCTC 42454T
KCTC 2346T
A
PE
PL
2nd
1st
PE
PG
PL
2nd
1st
PG
PE
PL
2nd
1st
PG
PE
PL
2nd
1st
B
PE
2nd
1st
PE
PL
2nd
1st
PE
PL
2nd
1st
PE
2nd
1st
C
2nd
1st
2nd
1st
2nd
1st
2nd
1st
D
L1
PE
SGL
PG
PL
2nd
1st
L1
SGL
PG
PE
PL
2nd
1st
L1
PE
PG
SGL
PL
2nd
1st
L2
L1
PG
PE
SGL
PL
2nd
1st

Figure S2. The two-dimensional thin-layer chromatograms of the polar lipids from AK-PDB1-5^{T} and related type strains detected with the following reagents: molybdenum blue (A)**,** ninhydrin (B), Dragendorff reagent (C), and phosphomolydbic acid (D). *Sphingomonas nostoxanthinifaciens* AK-PDB1-5^{T}; *Sphingomonas crusticola* MIMD3^{T} (=KCTC 42801^{T}); *Sphingomonas vulcanisoli* SN6-13^{T} (=KCTC 42454^{T}); *Sphingomonas paucimobilis* KCTC 2346^{T}. SGL, Sphingoglycolipid; PE, phosphatidylethanolamine; PG, phosphatidylglycerol; PL, phospholipids; L, lipids.

A

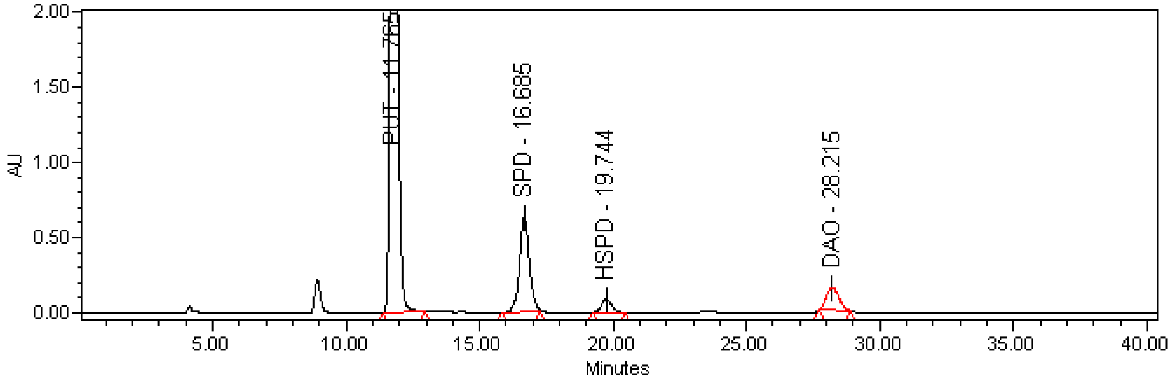

B

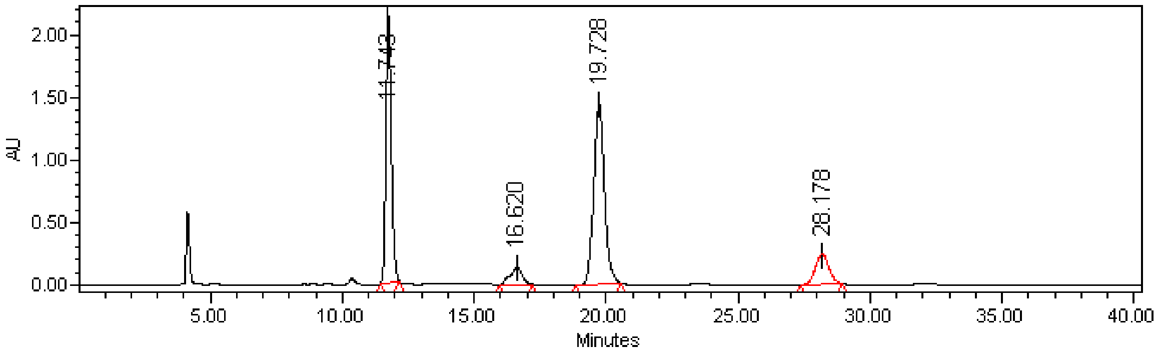

Figure S3. Analysis of polyamines in strain AK-PDB1-5^{T} by HPLC. A, Polyamine standard. B, Strain AK-PDB1-5^{T}. PUT, putrescine; SPD, spermidine; HSPD, homospermidine; DAO, diaminooctane.

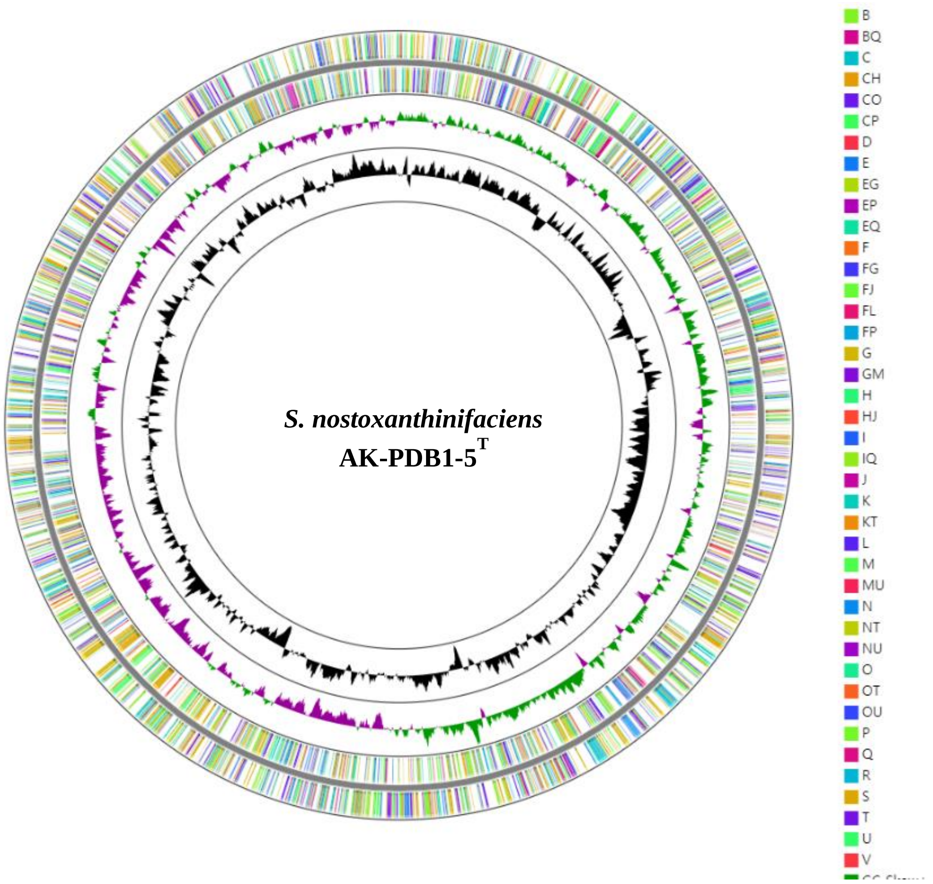

Figure S4. Map of the AK-PDB1-5^T genome generated with CGView. Marked characteristics are shown from the outside to the center. Rings 1 and 2 show cluster orthologous group (COG) annotation in the forward and reverse directions, respectively. Ring 3 shows the GC skew, while ring 4 shows the G+C % content plot. The COG categories are: A, RNA processing and modification; B, chromatin structure and dynamics; C, energy production and conversion; D, cell cycle control, cell division, and chromosome partitioning; E, amino acid transport and metabolism; F, nucleotide transport and metabolism; G, carbohydrate transport and metabolism; H, coenzyme transport and metabolism; I, lipid transport and metabolism; J, translation, ribosomal structure, and biogenesis; K, transcription; L, replication, recombination, and repair; M, cell wall/membrane/envelope biogenesis; N, cell motility; O, post-translational modification, protein turnover, chaperones; P, inorganic ion transport and metabolism; Q, secondary metabolite biosynthesis, transport, and catabolism; R, general function prediction only; S, function unknown; T, signal transduction mechanisms; U, intracellular trafficking, secretion, and vesicular transport; V, defense mechanisms; W, extracellular structures; X, mobilome: prophages, transposons; and Z, cytoskeleton.

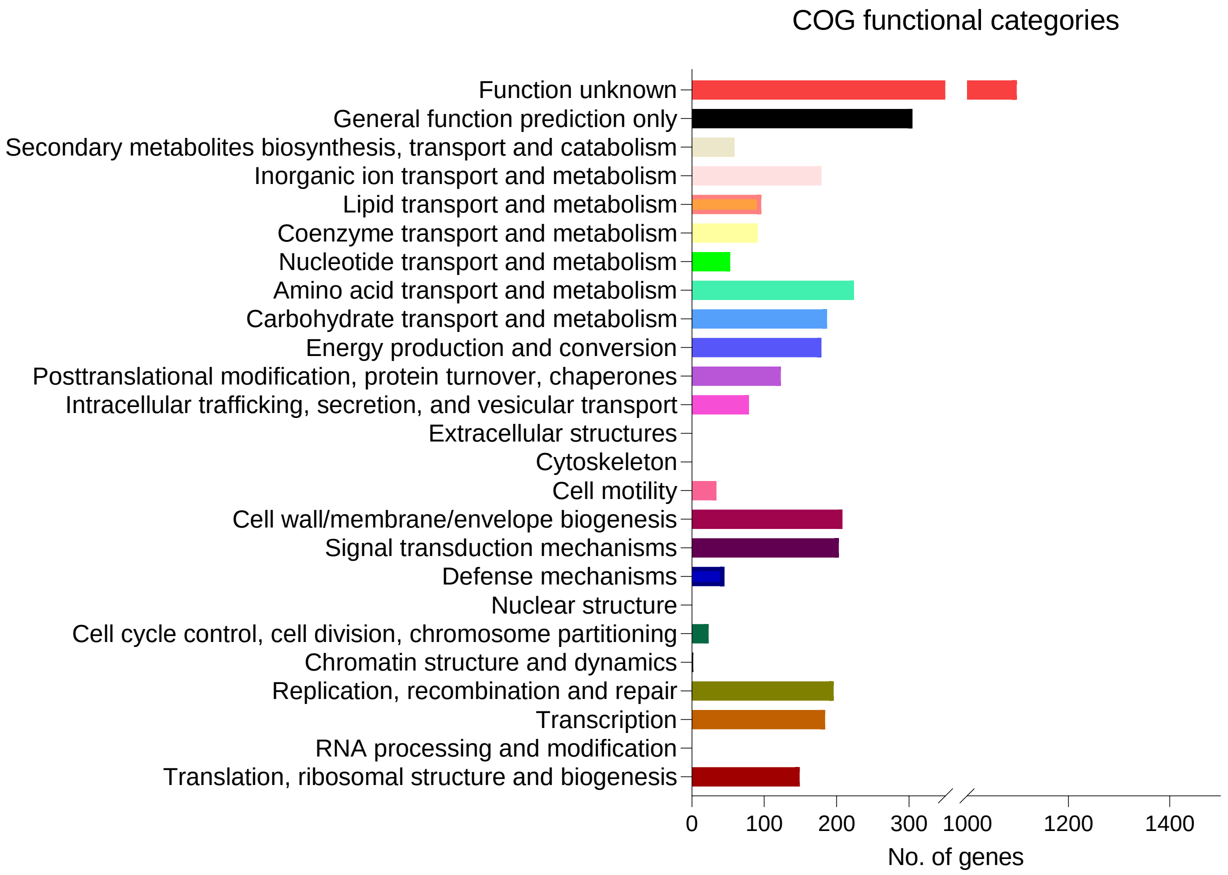

Figure S5. COG classification of strain AK-PDB1-5^T genome. A total of 4,040 transcripts were identified via the eggnog database: 3,717 of them were categorized into 25 COG clusters.

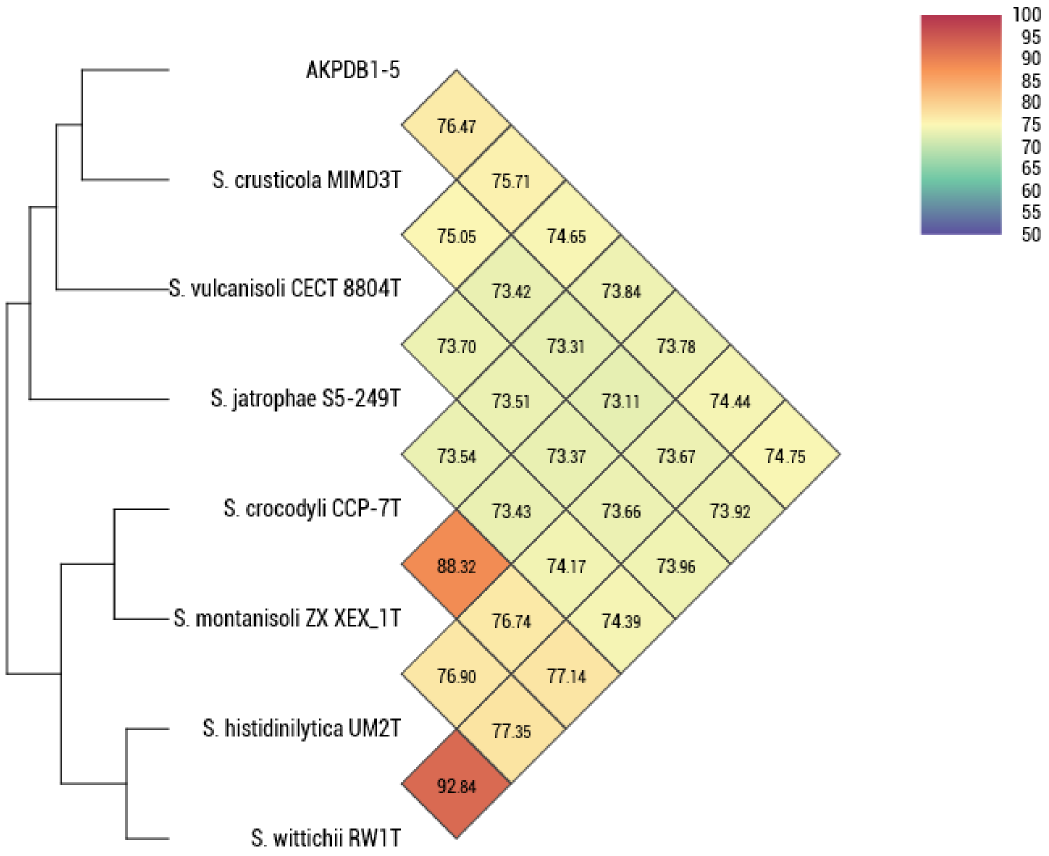

Figure S6. **Heatmap generated with OrthoANI value calculated using the OAT software, comparing AK-PDB1-5^{T} and closely related strains of *Sphingomonas*.** The color code indicates the closest species in red and the farthest in green. The unweighted pair group method with arithmetic mean (UPGMA) dendrogram based on the OrthoANI values of eight species is listed on the left.

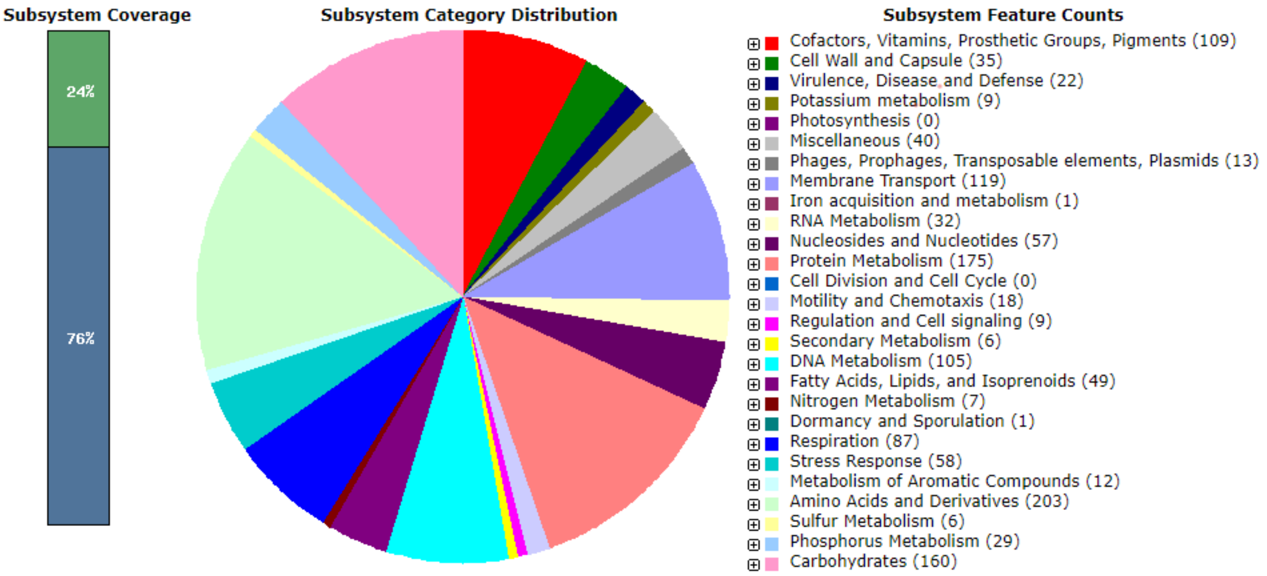

Figure S7. Distribution and count of the subsystem categories of strain AK-PDB1-5^{T} based on RAST (Rapid Annotation using Subsystem Technology) annotation.

A

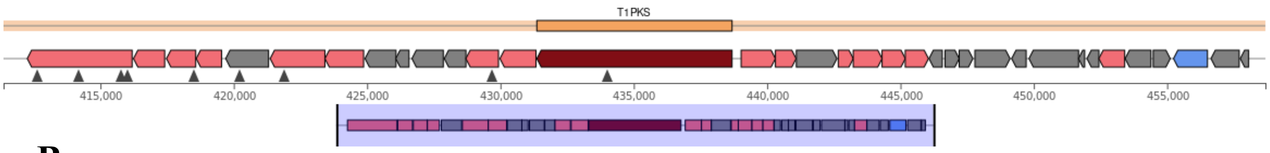

B

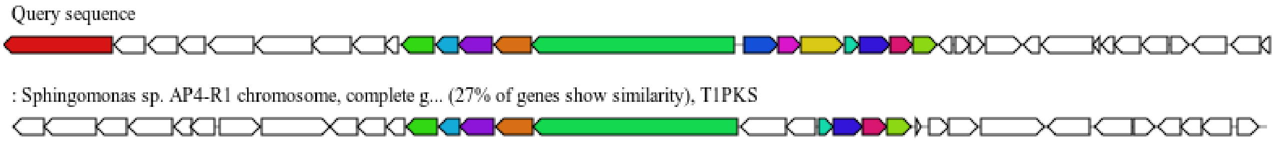

C

Contig no./locus tag	Identified protein
ctg1_388	Rhamnan synthesis protein F
ctg1_389	DegT/DnrJ/EryC1/StrS aminotransferase family
ctg1_390	gmd: GDP-mannose 4,6-dehydratase
ctg1_391	NAD dependent epimerase/dehydratase family
ctg1_392	No significant similarity
ctg1_393	Glycosyltransferase family 9 (heptosyltransferase)
ctg1_394	pfkB family carbohydrate kinase
ctg1_395	N-acylglucosamine 2-epimerase (GlcNAc 2-epimerase)
ctg1_396	Histidinol-ppas: histidinol-phosphate phosphatase domain
ctg1_397	No significant similarity
ctg1_398	ABC-2 type transporter
ctg1_399	Glycosyl transferases group 1
ctg1_400	Aminotransferase class I and II
ctg1_401	Beta-ketoacyl synthase, N-terminal domain
ctg1_402	polysaccharide biosynthetic process
ctg1_403	short chain dehydrogenase
ctg1_404	sulfuric ester hydrolase activity
ctg1_405	rmlC: dTDP-4-dehydrorhamnose 3,5-epimerase
ctg1_406	dTDP_gluc_dehyt: dTDP-glucose 4,6-dehydratase
ctg1_407	RmlD substrate binding domain
ctg1_408	rmlA: glucose-1-phosphate thymidylyltransferase
ctg1_409	SmpA / OmlA family
ctg1_410	Ubiquinol-cytochrome C chaperone
ctg1_411	Large ribosomal RNA subunit accumulation protein YceD
ctg1_412	Methyl-accepting chemotaxis protein (MCP) signalling domain
ctg1_413	Single-strand binding protein family
ctg1_414	Ferrous iron transport protein B

ctg1_415	transition metal ion binding
ctg1_416	nucleic acid binding
ctg1_417	Phosphotransferase enzyme family
ctg1_418	ispH_lytB: 4-hydroxy-3-methylbut-2-enyl diphosphate reductase
ctg1_419	No significant similarity
ctg1_420	Sodium:dicarboxylate symporter family
ctg1_421	L-rhamnose-proton symport protein (RhaT)
ctg1_422	L-rhamnose mutarotase

Figure S8. Information of type I polyketide (T1PKS) synthases biosynthetic gene cluster of AK-PDB1-5^{T} predicted via anti-SMASH. A, Identified secondary metabolite regions using strictness "relaxed" option for detection. B, Type I polyketide synthase TIPKS metabolite clusterblast compared with the NCBI database. C, Pfam-based GO term annotation gene. Maroon highlighting denotes core biosynthetic genes while pink highlighted rows shows additional biosynthetic genes.

A

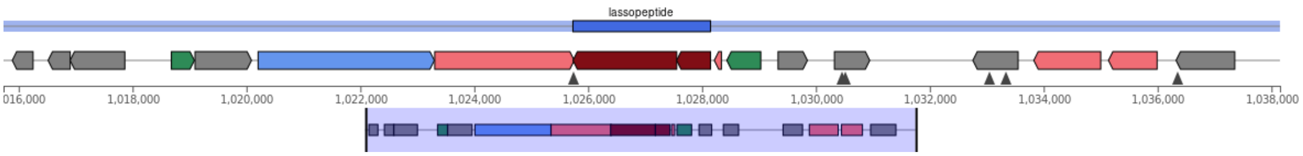

B

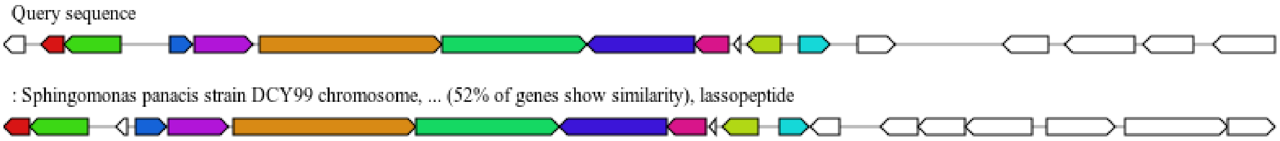

C

Contig no./locus tag	Identified protein
ctg1_923	NIPSNAP
ctg1_924	Protein of unknown function (DUF2958)
ctg1_925	Metallopeptidase superfamily domain
ctg1_926	Sigma70-ECF: RNA polymerase sigma factor, sigma-70 family
ctg1_927	FecR protein
ctg1_928	TonB-dependent siderophore receptor family
ctg1_929	Prolyl oligopeptidase family
ctg1_930	Asparagine synthase
ctg1_931	Transglutaminase-like superfamily
ctg1_932	No significant similarity
ctg1_933	Bacterial regulatory proteins, gntR family
ctg1_934	No significant similarity
ctg1_935	No significant similarity
ctg1_936	Putative MetA-pathway of phenol degradation
ctg1_937	Alcohol dehydrogenase GroES-associated
ctg1_938	NADH:flavin oxidoreductase / NADH oxidase family
ctg1_939	Transposase

Figure S9. Information of lasso peptide biosynthetic gene cluster of strain AK-PDB1-5^{T} predicted by antiSMASH. A, Identified secondary metabolite regions using strictness "relaxed" option for detection. B, Lasso peptide metabolite clusterblast compared with the NCBI database. C, Pfam-based GO term annotation of genes. Maroon highlighting denotes core biosynthetic genes, while pink highlighting shows additional biosynthetic genes.

A

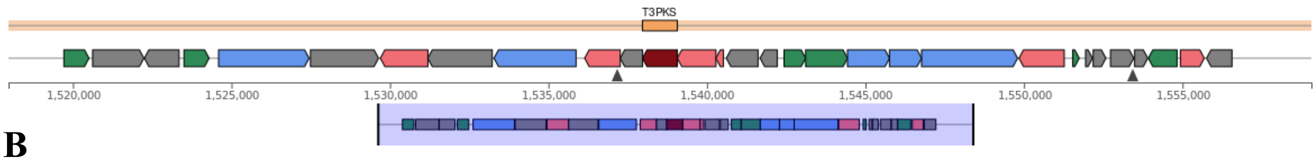

B

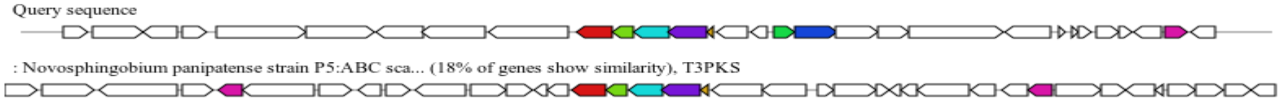

C

Contig no./locus tag	Identified protein
ctg1_1427	Bacterial regulatory proteins, gntR family
ctg1_1428	L-lactate permease
ctg1_1429	NHL repeat
ctg1_1430	IclR helix-turn-helix domain
ctg1_1431	TonB-dependent Receptor Plug Domain
ctg1_1432	Hypothetical glycosyl hydrolase 6
ctg1_1433	Tryptophan halogenase
ctg1_1434	No significant similarity
ctg1_1435	TonB-dependent Receptor Plug Domain
ctg1_1436	NAD(P)-binding Rossmann-like domain
ctg1_1437	Methyltransferase domain
ctg1_1438	Chalcone and stilbene synthases, N-terminal domain
ctg1_1439	Beta-ketoacyl synthase, N-terminal domain
ctg1_1440	Phosphopantetheine attachment site
ctg1_1441	No significant similarity
ctg1_1442	No significant similarity
ctg1_1443	Response regulator receiver domain
ctg1_1444	HAMP domain
ctg1_1445	Outer membrane efflux protein
ctg1_1446	HlyD family secretion protein
ctg1_1447	AcrB/AcrD/AcrF family
ctg1_1448	Peptidase family M20/M25/M40
ctg1_1449	Cold-shock' DNA-binding domain
ctg1_1450	No significant similarity
ctg1_1451	No significant similarity
ctg1_1452	No significant similarity
ctg1_1453	Uncharacterized protein family
ctg1_1454	Bacterial regulatory helix-turn-helix protein, lysR family

ctg1_1455	Enoyl-(Acyl carrier protein) reductase
ctg1_1456	No significant similarity

Figure S10. The BGCs type III polyketide synthases (T3PKS) obtained in strain AK-PDB1-5^{T} predicted via anti-SMASH. A, Identified secondary metabolite regions using strictness "relaxed". B, T3PKS metabolite clusterblast compared with the NCBI database. C, Pfam-based GO term annotation gene. Maroon highlighting denotes a core biosynthetic gene while pink highlighting shows additional biosynthetic genes.

A

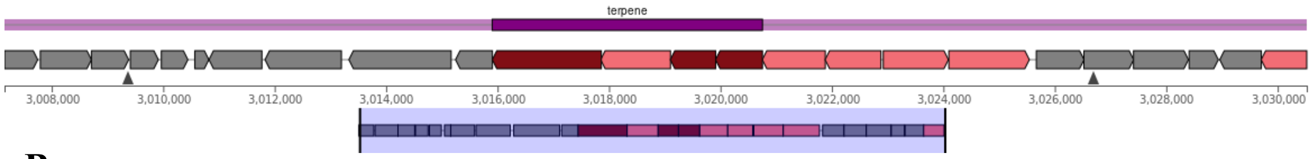

B

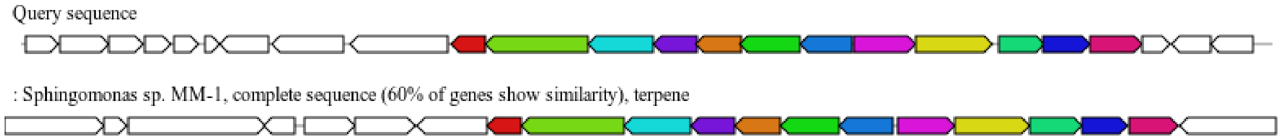

C

Contig no./locus tag	Identified protein
ctg1_2759	LemA family
ctg1_2760	TPM domain
ctg1_2761	No significant similarity
ctg1_2762	NUDIX domain
ctg1_2763	PilZ domain
ctg1_2764	No significant similarity
ctg1_2765	Aspartyl protease
ctg1_2766	DNA photolyase
ctg1_2767	Beta-L-arabinofuranosidase, GH127
ctg1_2768	Phosphorylase superfamily
ctg1_2769	Squalene-hopene cyclase N-terminal domain
ctg1_2770	Flavin containing amine oxidoreductase
ctg1_2771	Squalene/phytoene synthase
ctg1_2772	Squalene/phytoene synthase
ctg1_2773	Glycosyl transferase family 2
ctg1_2774	NAD dependent epimerase/dehydratase family
ctg1_2775	Glycosyl transferase family 21
ctg1_2776	Radical SAM superfamily
ctg1_2777	YdjC-like protein
ctg1_2778	No significant similarity
ctg1_2779	Lysylphosphatidylglycerol synthase TM region
ctg1_2780	Acetyltransferase (GNAT) family
ctg1_2781	Eukaryotic phosphomannomutase
ctg1_2782	Haloacid dehalogenase-like hydrolase

Figure S11. The BGCs terpene obtained in strain AK-PDB1-5^T was predicted via anti-SMASH. A, Identified secondary metabolite regions using strictness “relaxed”. B, Terpene metabolite clusterblast compared with the NCBI database. C, Pfam-based GO term annotation gene. Maroon highlighting denotes a core biosynthetic gene while pink highlighting shows additional biosynthetic genes.

A

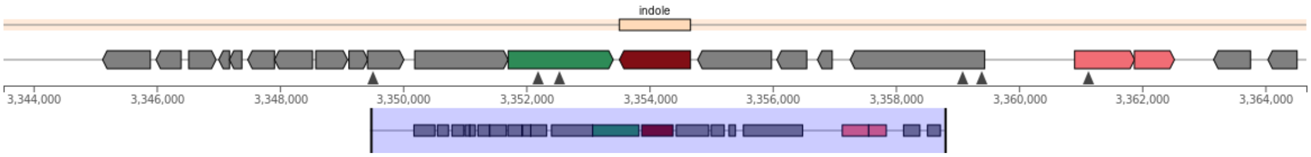

B

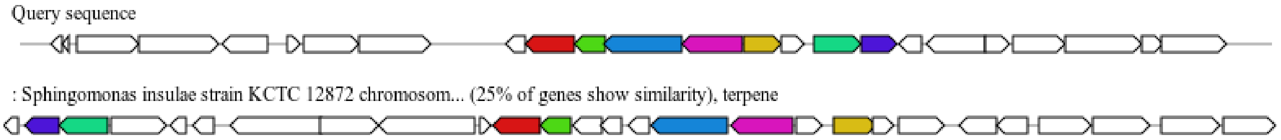

C

Contig no./locus tag	Identified protein
ctg1_3112	No significant similarity
ctg1_3113	Response regulator receiver domain
ctg1_3114	No significant similarity
ctg1_3115	Helix-hairpin-helix motif
ctg1_3116	No significant similarity
ctg1_3117	Protein of unknown function (DUF1810)
ctg1_3118	SOS response associated peptidase (SRAP)
ctg1_3119	SOS response associated peptidase (SRAP)
ctg1_3120	No significant similarity
ctg1_3121	2OG-Fe(II) oxygenase superfamily
ctg1_3122	KaiC
ctg1_3123	His Kinase A (phospho-acceptor) domain
ctg1_3124	Ferritin-like
ctg1_3125	Uncharacterized protein conserved in bacteria (DUF2252)
ctg1_3126	Domain of unknown function (DUF892)
ctg1_3127	No significant similarity
ctg1_3128	Catalase-related immune-responsive
ctg1_3129	No significant similarity
ctg1_3130	GlcNAc-PI de-N-acetylase
ctg1_3131	No significant similarity
ctg1_3132	Hsp20/alpha crystallin family

Figure S12. The BGCs indole obtained in strain AK-PDB1-5^{T} predicted via anti-SMASH. A, Identified secondary metabolite regions using strictness “relaxed”. B, Indole metabolite clusterblast compared with the NCBI database. C, Pfam-based GO term annotation gene. Maroon highlighting denotes a core biosynthetic gene while pink highlighting shows additional biosynthetic genes.

A

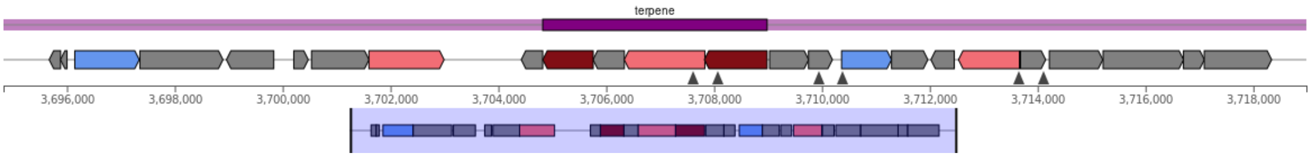

B

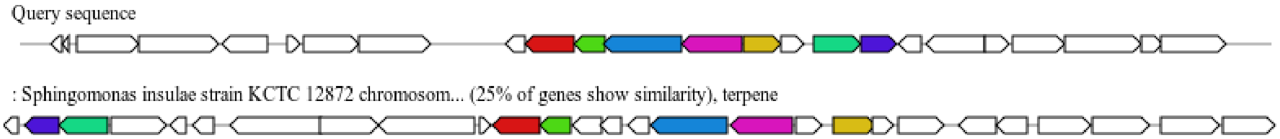

C

Contig no./locus tag	Identified protein
ctg1_3426	No significant similarity
ctg1_3427	No significant similarity
ctg1_3428	Major Facilitator Superfamily
ctg1_3429	Sodium/hydrogen exchanger family
ctg1_3430	Uncharacterized protein conserved in bacteria (DUF2171)
ctg1_3431	No significant similarity
ctg1_3432	Phytanoyl-CoA dioxygenase (PhyH)): [85:233]
ctg1_3433	Capsule polysaccharide biosynthesis protein
ctg1_3434	No significant similarity
ctg1_3435	Squalene/phytoene synthase
ctg1_3436	Possible lysine decarboxylase
ctg1_3437	Flavin containing amine oxidoreductase
ctg1_3438	Lycopene cyclase protein
ctg1_3439	Fatty acid hydroxylase superfamily
ctg1_3440	OsmC-like protein
ctg1_3441	EamA-like transporter family
ctg1_3442	COQ9
ctg1_3443	No significant similarity
ctg1_3444	FAD dependent oxidoreductase
ctg1_3445	Type IV leader peptidase family
ctg1_3446	SAF domain
ctg1_3447	Pilus formation protein N terminal region
ctg1_3448	Pilus biogenesis CpaD protein (pilus_cpaD)
ctg1_3449	No significant similarity

Figure S13. The BGCs zeaxanthin obtained in strain AK-PDB1-5^T was predicted via anti-SMASH. A, Identified secondary metabolite regions using strictness “relaxed”. B, Terpene (zeaxanthin) metabolite clusterblast compared with the NCBI database. C, Pfam-based GO term annotation gene. M Maroon highlighting denotes a core biosynthetic gene while pink highlighting shows additional biosynthetic genes.

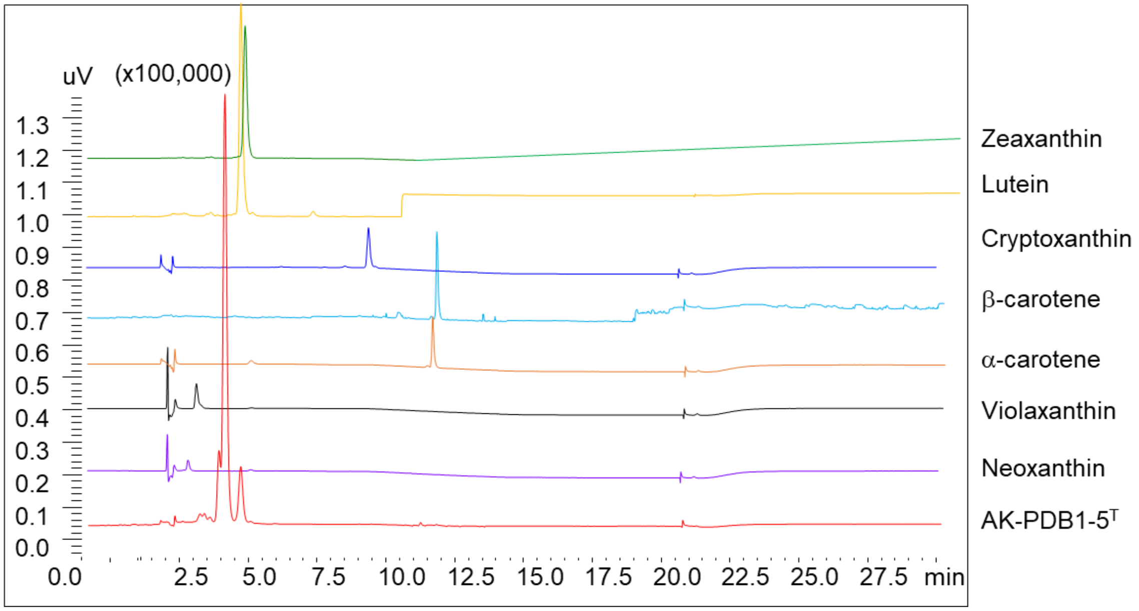

Figure S14. HPLC elution profiles of the pigments extracted from strain AK-PDB1-5^T. Zeaxanthin, lutein, cryptoxanthin, β-carotene, α-carotene, violaxanthin, and neoxanthin were included as carotenoid standards.

Table S1. The genome features of all type species in the genus *Sphingomonas* used in this study.

Type species	N_{50}	Completeness	Contamination	Quality	NCBI assembly number	Genome length (Mbp)	DNA G+C content (%)	Number of contigs	Total genes	Protein coding genes	Genes (RNA)	rRNAs (5S, 16S, 23S)	tRNA genes	ncRNAs	Pseudo Genes
S. nostoxanthinifaciens AK-PDB1-5^T	4,298,284	100%	1.9%	90.5 %	GCF_019930585.1	4,298,284	67.0	1	4139	4032	60	2, 2, 2	50	4	47
S. montana W16RD^T	202,237	100%	0.9%	95.5 %	GCF_001956315.1	3,722,743	67.0	35	3457	3352	51	1, 1, 1	45	3	54
S. prati CGMCC 1.15645^T	559,893	100%	0.9%	95.5 %	GCF_014643515.1	3,803,795	67.0	24	3477	3379	52	1, 1, 1	46	3	46
S. profundi LMO-1^T	4,538,634	100%	0%	100 %	GCF_009739515.1	4,538,634	69.2	1	4332	4181	56	2, 2, 2	47	3	95
S. solaris R4DWN^T	4,538,634	100%	0.9%	95.5 %	GCF_007785815.1	4,444,219	67.9	229	4294	4088	52	1, 1, 1	46	3	154
S. jatrophae S5-249^T	1,320,977	100%	0.9%	95.5 %	GCF_900113315.1	4,060,565	68.5	6	3887	3804	60	3, 2, 3	49	3	23
S. crusticola MIMD3^T	3,366,072	100%	0%	100 %	GCF_003391115.1	3,367,145	64.7	2	3195	3118	52	1, 1, 1	45	4	25
S. vulcanisoli CECT 8^T	423,091	99.1 %	1.9 %	89.6	GCF_011761305.1	3,267,440	64.6	17	3252	3113	51	1, 1, 1	45	3	88
S. oligoaromativorans DSM 102246^T	434,306	100 %	0.9 %	95.5	GCF_011762195.1	3,867,692	67.4	27	3729	3600	58	1, 2, 1	51	3	71
S. sanxanigenens DSM 19645^T	6,205,897	99.1 %	2.8 %	85.1	GCF_000512205.2	6,580,29	66.7	2	6246	6066	69	3, 3, 3	57	3	111

S. gilva ZDH117^{T}	850,722	99.1 %	1.9 %	89.6	GCF_003515075.1	3,541,169	67.6	17	3406	3335	51	1, 1, 1	45	3	20
S. crocodyli CCP-7^{T}	3041622	95.5 %	0.9 %	91	GCF_004005865.1	4,883,533	64.5	8	4713	4625	54	1, 1, 1	48	3	35
R. dicambivorans Ndbn-20^{T}	5026772	95.5 %	0.9 %	91	GCF_002355275.1	5,541,299	65.5	1	5295	5133	58	2, 2, 2	49	3	104
R. wittichii RW1^{T}	5,382,261	95.5%.	0.0%	95.5	GCF_000016765.1	5,915,246	67.9	3	4941	4850	56	2, 2, 2	48	2	35
R. histidinilytica UM2 ^{T}	409,683	95.5 %	0.0%	95.5	GCF_900167915.1	5,473,339	67.4	59	5288	5191	53	1, 1, 1	47	3	44
S. oleivorans FW-11^{T}	464,715	100 %	2.8 %	86	GCF_003050615.1	3,873,878	65.4	19	3671	3532	53	1, 1, 1	47	3	86
S. formosensis CC-Nfb-2^{T}	503,530	99.1 %	0.9 %	94.6	GCF_009755815.1	6,899,075	65.2	39	6408	6235	59	2, 5, 2	47	3	115
S. laterariae LNB2^{T}	136,374	92.5 %	0.9 %	88	GCF_900188165.1	4,419,388	65.4	85	4353	4231	53	1, 1, 1	47	3	69
S. haloaromaticamans P3^{T}	638,771	97.2 %	2.8 %	83.2	GCF_001853345.1	4,485,253	73.0	14	4719	4572	57	2, 2, 2	48	3	90
S. fennica K101^{T}	97,465	100 %	0.9 %	95.5	GCA_003034225.1	4,494,276	66.8 %	89	4443	4313	52	1, 1, 1	46	3	78

Table S2. Presence of secondary metabolite biosynthetic gene clusters in the genome sequence of strain AK-PDB1-5^{T}, as detected using antiSMASH.

Type	From	To	smBGC type	Most similar known cluster	Similarit y	Core biosynthe tic gene	Additional biosynthetic gene
T1PKS	411,366	458,685	Sphingan polysaccharide	Saccharide	17%	1	0
Lassopeptide	1,015,733	1,038,149	-	-	-	2	0
T3PKS	1,517,967	1,559,067	-	-	-	1	0
Terpene	3,007,152	3,030,521	Malleobactin A / malleobactin B / malleobactin C / malleobactin D	NRP:NRP siderophore	7%	3	1
Indole	3,343,512	3,364,666	-	-	-	1	0
Terpene	3,694,823	3,718,983	Zeaxanthin	Terpene	100%	2	1
